# Predicting risk of preterm birth in singleton pregnancies using machine learning algorithms

**DOI:** 10.3389/fdata.2024.1291196

**Published:** 2024-02-29

**Authors:** Qiu-Yan Yu, Ying Lin, Yu-Run Zhou, Xin-Jun Yang, Joris Hemelaar

**Affiliations:** ^1^National Perinatal Epidemiology Unit, Nuffield Department of Population Health, University of Oxford, Oxford, United Kingdom; ^2^Department of Preventive Medicine, School of Public Health, Wenzhou Medical University, Wenzhou, China; ^3^Wenzhou Women and Children Health Guidance Center, Wenzhou, China

**Keywords:** preterm birth, machine learning, prediction models, antenatal care, feature selection

## Abstract

We aimed to develop, train, and validate machine learning models for predicting preterm birth (<37 weeks' gestation) in singleton pregnancies at different gestational intervals. Models were developed based on complete data from 22,603 singleton pregnancies from a prospective population-based cohort study that was conducted in 51 midwifery clinics and hospitals in Wenzhou City of China between 2014 and 2016. We applied Catboost, Random Forest, Stacked Model, Deep Neural Networks (DNN), and Support Vector Machine (SVM) algorithms, as well as logistic regression, to conduct feature selection and predictive modeling. Feature selection was implemented based on permutation-based feature importance lists derived from the machine learning models including all features, using a balanced training data set. To develop prediction models, the top 10%, 25%, and 50% most important predictive features were selected. Prediction models were developed with the training data set with 5-fold cross-validation for internal validation. Model performance was assessed using area under the receiver operating curve (AUC) values. The CatBoost-based prediction model after 26 weeks' gestation performed best with an AUC value of 0.70 (0.67, 0.73), accuracy of 0.81, sensitivity of 0.47, and specificity of 0.83. Number of antenatal care visits before 24 weeks' gestation, aspartate aminotransferase level at registration, symphysis fundal height, maternal weight, abdominal circumference, and blood pressure emerged as strong predictors after 26 completed weeks. The application of machine learning on pregnancy surveillance data is a promising approach to predict preterm birth and we identified several modifiable antenatal predictors.

## Introduction

Preterm birth (PTB) is the leading cause of neonatal and child mortality globally (Liu et al., 2016). United Nations Sustainable Development Goal 3 target 3.2 aims to reduce neonatal and child mortality to 12 per 1,000 live births and 25 per 1,000 live births, respectively (United Nations, 2016). A recent study estimated that 10.6% of all babies worldwide are born prematurely, with Asia accounting for 7.84 million (52.9%) PTBs. In particular, China accounts for an estimated 1.17 million PTBs annually, highlighting an urgent public health issue (Chawanpaiboon et al., 2019).

Early detection of pregnant women at risk of preterm birth helps high-risk pregnant women to receive timely preventative interventions to reduce the risk of PTB (ACOG, 2021). Imaging tests or invasive screening have potential as effective screening methods, but remain experimental because of high cost, possible harm, and low accessibility (Bahado-Singh et al., 2019; Considine et al., 2019; Wang et al., 2019). Non-invasive screening measures using machine learning (ML) algorithms based on large-scale pregnancy surveillance data with multilevel information linkage to delivery records promises to be beneficial to support clinical decision making to predict adverse pregnancy outcomes and guide pregnancy management without any extra physiological or imaging tests (Gao et al., 2019; Sharifi-Heris et al., 2022).

Prediction models using ML algorithms to quantify the risk of PTB have been proposed in recent years, with predictive powers ranging from 0.6 to 0.9 (Weber et al., 2018; Koivu and Sairanen, 2020; Arabi Belaghi et al., 2021; Raja et al., 2021; Shields et al., 2021; Lee et al., 2022; Nieto-Del-Amor et al., 2022; Sun et al., 2022). Some ML prediction models using uterine electrohysterographic (EHG) signals and multi-omics in the middle trimester reported a good ability to differentiate between preterm and term birth (Tarca et al., 2021; Mohammadi Far et al., 2022; Nieto-Del-Amor et al., 2022; Romero-Morales et al., 2022; Espinosa et al., 2023). However, these predictors are time consuming and costly, and are impossible to measure in routine antenatal care in low-resource settings. Instead, prediction models using maternal features available from routine pregnancy care are more likely to be widely applicable and improve pregnancy outcomes. To improve predictive power of PTB, many popular ML algorithms have been employed and compared with traditional regression methods and achieved high areas under the receiver operating characteristic curve (AUC) (Fazzari et al., 2022; Park et al., 2022; Nsugbe et al., 2023). A number of studies found that logistic regression provided quicker and better classification performance, and easier interpretability than ML models in other disease settings (Kuhle et al., 2018; Song et al., 2023). However, a study comparing deep learning with logistic regression found that neural networks showed slightly better predictive power of PTB than logistic regression (Goldsztejn and Nehorai, 2023). To achieve an efficient prediction model, feature selection is an important process to reduce dimensionality and computing complexity, and facilitate clinical practice. There are two conventional ways to conduct feature selection: one is applying univariate analysis to select features which are highly associated with the outcome (Park et al., 2022; Nsugbe et al., 2023), another is relying on feature importance derived from ML algorithms (Sharifi-Heris et al., 2022; Espinosa et al., 2023). However, some known important features might be ignored when only relying on ML-based feature importance lists (Bose et al., 2019; Liverani et al., 2023). Moreover, predictive models are at risk of overestimation or underestimation bias, due to inappropriate data sources (Sun et al., 2022), confounding factors as predictors (Raja et al., 2021), poor definition of predictors (Lee et al., 2022), incomplete reporting of modeling processes (Shields et al., 2021; Lee et al., 2022; Sun et al., 2022), inappropriate statistical approaches to perform feature selection (Weber et al., 2018; Koivu and Sairanen, 2020; Arabi Belaghi et al., 2021), and absence of handling of imbalanced data (AlSaad et al., 2022; Fazzari et al., 2022).

To overcome the limitations of previous studies we designed and utilized detailed methodology to perform data pre-processing and select predictors using feature importance derived from the ML algorithms, combined with clinical knowledge. We aimed to develop and validate PTB prediction models at different gestational intervals to support application in clinical practice.

## Materials and methods

### Study design and population

A prospective population-based cohort study was conducted in 51 midwifery clinics and hospitals in Wenzhou City located in Zhejiang Province of China, recruiting 355,062 pregnant women at around 12-week gestation. We included all singleton pregnancies who delivered at < 42 weeks' gestation from 1 January 2014 to 31 December 2016. Exclusion criteria were absence of follow-up antenatal records or birth records, multiple pregnancy (e.g, twins), missing values of any features listed in Supplementary Table S1, and deliveries at < 24 weeks with birthweight over 1000 g, deliveries at > 24 weeks but with weight Z scores beyond the range of −3 and 3 according to Intergrowth 21th standard for newborn weight (Supplementary Figure S1) (Villar et al., 2014). Supplementary Figure S2 shows the selection process of participants, and a total of 22,603 singleton pregnancies with complete data were included in the analysis. The data sets were de-identified and we were authorized to access these datasets. The study was approved by the ethics committee of the Second Hospital Affiliated to Wenzhou Medical University.

### Outcomes

PTB was defined as birth occurring between 24 and 36 + 6 weeks' gestation, regardless of whether the PTB was spontaneous or medically indicated. The gestational age at birth was determined by ultrasound estimation at the first antenatal care visit.

### Data collection

The Wenzhou maternal and child health information management platform was used to collect health records of pregnancy health care before, during, and after delivery. At registration, each pregnant woman was recruited and interviewed using a standardized questionnaire to gather demographic and lifestyle information, pregnancy history, and medical history by a trained obstetric doctor, and laboratory tests were taken after fasting overnight. We collected information, including maternal age, height, weight, education, occupation, and ethnicity. Further, we included parity, maternal heart rate, gynecological history, and clinically confirmed disease history, behaviors (smoking, medicine use, alcohol use and contraception) in the last 3 months, menstruation (length of menstrual cycle, length of a menstrual period, age at menarche). During antenatal care visits, vital signs, including blood pressure, Maternal Abdominal Circumference (MAC), Symphysis Fundal Height (SFH), and Systolic Blood Pressure (SBP), Diastolic Blood Pressure (DBP) and weight were measured by a designated obstetric doctor, and other additional laboratory tests if necessary. Birth records were linked to the antenatal care database and registration database. To increase robustness for further prediction modeling, rare features, including smoking, alcohol use, medicine use and contraception, that occurred in fewer than 1% of women were removed.

Pregnancy-associated laboratory tests at registration, tested after an overnight fast of more than 8 hours, were extracted as potential markers of PTB. These tests comprised hemoglobin, leukocyte count, platelet count, Fasting Blood Glucose (FBG), Alanine Aminotransferase (ALT), Aspartate Aminotransferase (AST), Albumin (AIB), Total Bilirubin (TBil), Serum creatinine (Scr), serum Urea Nitrogen (BUN), urine acetone bodies, Urine Occult Blood (ERY), Urine White Blood Cells (LEU), Urine Glucose (UGLU), and blood type. The features ERY, LEU, UGLU, and blood type were removed as they had >50% missing values.

Given the percentiles of gestational weeks at the first antenatal visit (Supplementary Table S2), the pregnancy period before 37 gestational weeks was divided into early pregnancy (< 18 weeks), middle of pregnancy (18 to 25+ 6 weeks), and late pregnancy (26 to 36 + 6 weeks). Antenatal measurements were also encoded in line with the gestational intervals. For example, if there were two antenatal visits before 18 weeks, the feature of SBP1 was created and assigned by averaging the two SBP values. The difference in SBP, DBP, SFH, MAC, and maternal weight between pregnancy periods was calculated to represent the absolute change between pregnancy periods. Supplementary Table S1 lists all 49 maternal features included as candidate predictors of PTB.

### Statistical methodology

**Figure 1:**
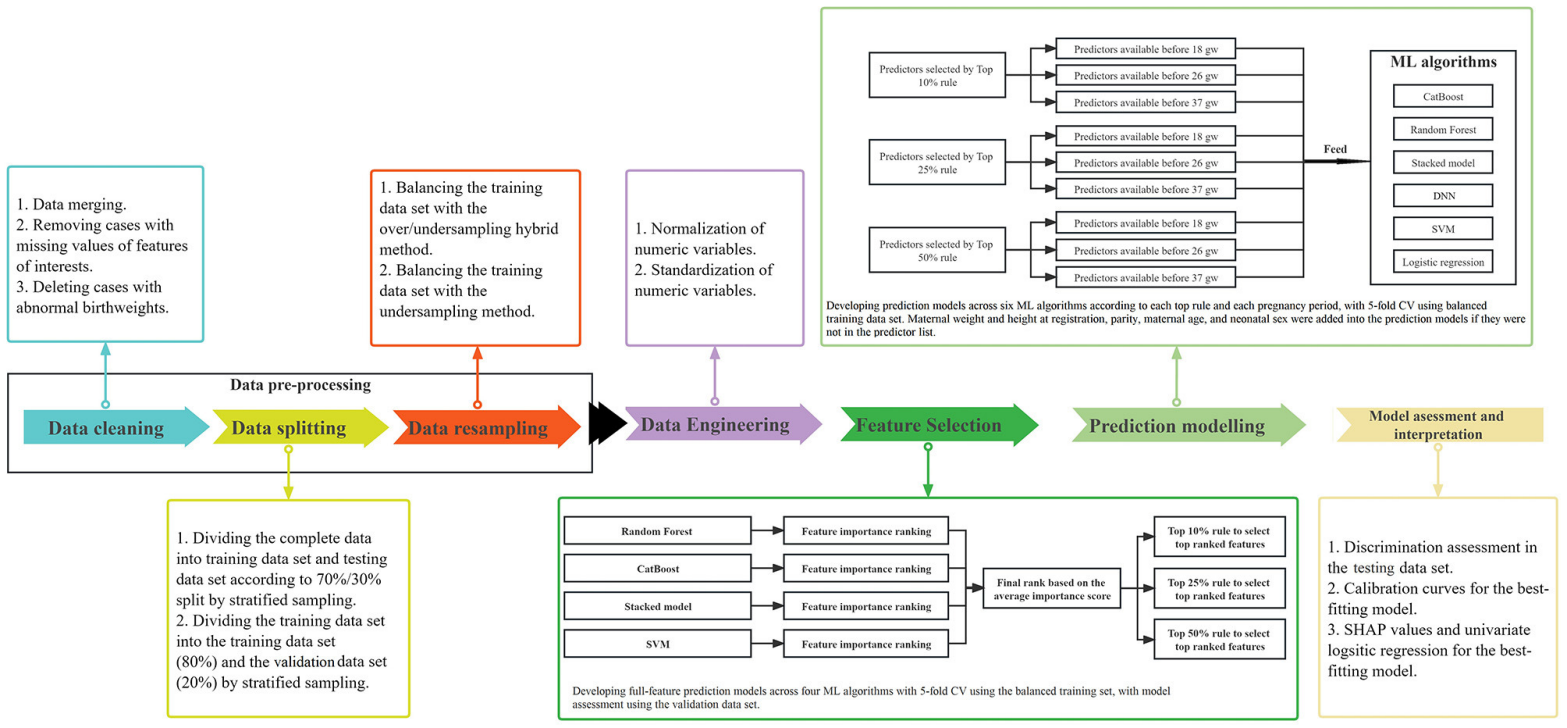
Diagram of the statistical methodology. ML, machine learning; CV, cross validation; gw, gestational weeks.

As illustrated in Figure 1, the process to construct models for predicting PTB was performed in a number of steps:

#### Data cleaning, splitting, and resampling

Firstly, data cleaning involved data merging, removing cases with missing values of features of interests, and deleting cases with abnormal birthweights. Secondly, we divided our data of 22,603 pregnancies with complete data into training and testing data sets according to a 70%/30% split, using stratified sampling. The training data set was then further divided into training (80%) and validation data sets (20%), using stratified sampling. The training data set was used to implement feature selection and develop the final prediction models with 5-fold cross validation and hyperparameter tuning. The validation data set was used to assess the performance of full-feature prediction models that were used to select a subset of optimal features. The testing data set was created to assess the performance of prediction models developed using the training data set with selected optimal features. Thirdly, to our knowledge, data involving singleton pregnancies always contain < 10% preterm births, which are imbalanced data that lead to decreased prediction performance using ML approaches. With imbalanced data, prediction models tend to favor the majority class as outcome to achieve high accuracy. Thus, we applied the over/undersampling hybrid method and the K-nearest neighbors for the undersampling method to balance the training data set, while keeping the testing and validation data sets imbalanced (Zhang et al., 2010; Nieto-Del-Amor et al., 2022). In our study, with the resampling methods, the training data set was resamplied into balanced data, resulting in preterm births and term births each occupying 50%, respectively.

#### Data engineering

Normalization (minimizing the skewedness of numeric variables by the Yeo-Johnson method) and standardization (centering and scaling numeric variables with zero mean and unit variance) of all continuous features was performed to improve model performance for the training, testing, and validation data sets (Boehmke and Greenwell, 2019; Raju et al., 2020).

#### Algorithms

Random Forest (Biau, 2012), CatBoost (Prokhorenkova et al., 2018; Wang et al., 2018), Support Vector Machine (SVM) (Noble, 2006; Gao et al., 2019), and Stacked Models (Van der Laan et al., 2007) were used to construct prediction models with all features to obtain permutation-based feature importance lists for feature selection. These four algorithms plus the Deep Neural Network (DNN) and logistic regression were used to develop PTB prediction models. The prediction models based on the balanced training data set were developed with 5-fold cross validation (CV). The full-feature prediction models for feature selection were validated in the validation data set, and the final prediction models with optimal subsets of features were validated in the testing data set.

Random Forest, an improvement over bagging decision trees, is an ensemble learning algorithm that produces successive independent trees fitted on bootstrapped random subsets of data (Biau, 2012). It creates additional randomness to select predictors among a random subset of features by splitting nodes. The bagging method combines multiple decision trees to achieve a more accurate and stable result (Biau, 2012).

CatBoost comprises one of the most efficient gradient boosting of decision trees algorithms, taking advantage of automatical handling of categorical features and missing values in the dataset to decrease overfitting. Compared to other gradient boosting algorithms such as XGBoost, it structures symmetric decision trees to enable efficient CPU (Central Processing Unit) implementation, reducing time-consumption, and acting as a regulator to improve overfitting (Prokhorenkova et al., 2018).

SVM is a very effective supervised machine learning algorithm which finds an optimal hyperplane based on multidimensional data to act as a class boundary to separate cases into different classes (Noble, 2006). The hyperplane strives to achieve a maximum margin between the closest points of different classes. The SVM with radial basis function kernel is used to implement predictive modeling.

Stacked Model, also called super learner, is an ensemble algorithm that stacks multiple traditional ML base learners such as the random forests and gradient boosting to find the optimal combination of diverse learning algorithms that make a prediction as-good-as or better than any single ML model (Polley and Van Der Laan, 2010). We applied generalized linear model, Deep Learning, Random Forests, XGBoost, Gradient Boosting Machine as base learners, and used the generalized linear model with non-negative weights to implement the ensemble process of base learners with “h2o” package in R software.

DNN, a type of deep learning, provide a multi-layer neural network to learn data as data sets have numeric dimensions of the features (Chollet and Allaire, 2018). DNN have widespread applications in image classification and voice recognition (Moreira et al., 2018; Chen and Xu, 2020). The feed-forward DNN we used here are densely connected layers where inputs impact on each successive layer which then affect the final output layer. To build a feed-forward DNN, we defined a network architecture with 4 hidden layers with the nodes ranging from 16 to 128, followed by an output layer with 2 nodes. Each hidden layer is activated by a Rectified linear unit (ReLU) function that is taking the summed weighted inputs in a previous layer and transforming them to a 0 (not fire) or > 0 (fire) if there is enough signal, and we used the sigmoid activation function for the output layer. A binary cross-entropy loss function and an optimizer of keras were established to assess the DNN accuracy and automatically adjust the weights across all the node connections to improve the overall predictive accuracy. The specific hyper-parameter set is shown in Supplementary Table S3.

Logistic regression (LR) is one of the most common statistical analysis models for predicting the probabilities of binary responses. Using the model equations, maximum likelihood estimation estimates the parameters of a probability distribution.

#### Feature selection

Four ML algorithms were applied to construct 49-feature prediction models which were employed to calculate permutation-based feature importance that were used to generate feature ranking lists (Altmann et al., 2010). The average importance score of each feature was calculated by the sum of the rankings across the four models divided by four, which was used to get the final ranking for each feature. The discrimination performance of 49-feature prediction models was validated in the validation data set. To achieve a model with the fewest predictors and best predictive power, the number of candidate predictors were selected according to the top 10%, top 25%, and top 50% rules based on the final rank list. 49-feature prediction models for feature selection, as well as the final prediction models according to the different rules of selecting the most important features, using the training data set, were all tuned by the random grid search strategy for hyper-parameters (Supplementary Table S3).

#### Predictive modeling

Under each rule of selecting the number of candidate predictors, five additional predictors of maternal weight and height at registration, parity, maternal age, and neonatal sex were added into the prediction models if they were not in the predictor list, considering their crucial contributions to perinatal health (Gardosi et al., 2018). Three sequential prediction models were developed to discriminate preterm birth from term birth, according to stage of pregnancy: early pregnancy models aimed to predict risk of PTB with data available before 18 weeks; Middle pregnancy models were constructed to evaluate the risk of preterm birth with data available before 26 weeks; Late pregnancy models are built to assess the risk of PTB after 26 weeks. Five–fold CV accuracy was used in the balanced training data set to assess model performance for internal validation.

#### Performance assessment and interpretation

For each ML predictive model developed with the balanced training set, we assessed the AUC value, accuracy (*Acc*) (Equation 1), sensitivity (*Sen*) (Equation 2), specificity (*Spec*) (Equation 3) using optimal threshold values in the validation data set. The optimal threshold value of receiver operating characteristic (ROC) curve was assigned as the point closest to the true positive rate of 1 and false positive rate of 0. The *Acc, Sen*, and *Spec* are computed as follows:


(1) $$\begin{array}{l} Acc=\frac{TP+TN}{TP+TN+FP+FN} \end{array}$$

(2) $$\begin{array}{l} Sen=\frac{TP}{TP+FN} \end{array}$$

(3) $$\begin{array}{l} Spec=\frac{TN}{TN+FP} \end{array}$$

*TP* and *FN* refer to the numbers of true positives (PTB classified as PTB) and false negatives (PTB classified as term birth), respectively. *TN* and *FP* refer to the numbers of true negatives (term births classified as term births) and false positives (term births classified as PTB), respectively.

We selected the best-fitting model with the highest AUC values, and the highest accuracy in cases of similar AUC values (± 0.02) in the testing data set. Calibration curves were plotted for the final optimal predictive model, developed with the balanced training set, to show predicted vs. observed outcomes with the testing set.

To provide interpretation for the best-fitting model, we applied Shapley Additive Explanations (SHAP) values to evaluate each predictive feature using the testing data set (Williamson and Feng, 2020). As a tool for visualizing the effect of individual features on the model results, SHAP values enable clinical practitioners to distinguish the key factors contributing to the risk of disease. The odds ratio (*OR*) and 95% confidential interval (*CI*) were calculated using univariate logistic regression in the cohort of 22,603 pregnancies to indicate the association of the predictors derived from the best-fitting model with PTB.

#### Software used

Data pre-processing was conducted in R (version 4.3.1). DNN models were run in Python (version 3.1, using “tensorflow” and “keras” packages), while the other ML models were run in R (version 3.6.1) (“h2o” package for random forests and stacked model; “catboost” package for CatBoost; “e1071” package for SVM). Cross validation was performed with the “caret” package, and SHAP values were calculated using the “fastshap” package. The ROC-AUC curves were plotted by the “pROC” and “ROCR” packages.

## Results

### Maternal characteristics at registration

After matching registration data, antenatal visit data, and birth data, 225,523 singleton pregnancies had health records in each of the three data resources (Supplementary Figure S2). After removing pregnancies with missing values of features of interests (*n* = 202,873) and births with abnormal birthweight (*n* = 47), 22,603 singleton pregnancies with 946 (4.2%) PTBs were retained. Table 1 shows the maternal features, including demographics, laboratory tests, and clinical history, collected at registration.

**Table 1:** Maternal characteristics in 22,603 singleton pregnancies in the complete data set.

**Features**	**Full-term birth**	**Preterm birth**	*P*-values^*^
No.	21,657	946	
No. of antenatal visits, median (P25, P75)	9 (7, 11)	7 (5, 8)	< 0.001
No. of antenatal visits before 24 gestational weeks, median (P25, P75)	2 (1, 3)	1 (0.2)	< 0.001
Ultrasound gestational weeks at the first visit, median (P25, P75)	16.00 (13.00, 17.00)	16.00 (13.00, 17.00)	0.055
Gestational age at childbirth, median (P25, P75)	39.43 (38.71, 40.14)	36.00 (34.86, 36.57)	< 0.001
**Occupation at registration**, ***n*** **(%)**			0.611
Farmer or fishermen	8,259 (38.1)	377 (39.9)	
Employee	2,645 (12.2)	107 (11.3)	
Business and service industry	2,478 (11.4)	113 (11.9)	
Households	5,642 (26.1)	230 (24.3)	
Others	2,633 (12.2)	119 (12.6)	
**Education**, ***n*** **(%)**			0.045
Primary school and below	965 (4.5)	56 (5.9)	
Secondary school and high school	13,273 (61.3)	590 (62.4)	
College and above	7,419 (34.3)	300 (31.7)	
Gynecologic surgery history, *n* (%)	872 (4.0)	64 (6.8)	< 0.001
Maternal complications, *n* (%)	69 (0.3)	21 (2.2)	< 0.001
Maternal age at registration, median (P25, P75), years	26.00 (24.00, 30.00)	27.00 (24.00, 31.00)	< 0.001
Age at menarche, median (P25, P75), years	14.00 (13.00, 15.00)	14.00 (13.00, 15.00)	0.404
Length days of a menstrual cycle, median (P25, P75), days	30.00 (28.00, 30.00)	30.00 (28.00, 30.00)	0.003
Days for a menstrual period, median (P25, P75), days	5.00 (4.00, 6.00)	5.00 (4.00, 6.00)	0.002
**Parity**, ***n*** **(%)**			< 0.001
0	12,084 (55.8)	470 (49.7)	
1	9,159 (42.3)	448 (47.3)	
>1	414 (1.9)	28 (3.0)	
Maternal height, median (P25, P75), cm	160.00 (156.00, 163.00)	158.00 (155.00, 162.00)	< 0.001
Maternal weight, median (P25, P75), kg	52.00 (48.00, 57.00)	52.50 (48.00, 58.00)	0.086
Maternal hear rate, median (P25, P75), times per minute	80.00 (75.00, 85.00)	80.00 (75.00, 85.00)	0.762
Hemoglobin, median (P25, P75), 10^9/*L*^	124.00 (118.00, 131.00)	125.00 (119.00, 132.00)	0.001
Leukocyte, median (P25, P75), 10^9/*L*^	7.90 (6.80, 9.30)	8.10 (7.00, 9.50)	0.002
Platelet, median (P25, P75), 10^9/*L*^	214.00 (185.00, 246.00)	222.00 (189.00, 254.75)	< 0.001
FBG, median (P25, P75), mmol/L	4.67 (4.40, 4.97)	4.69 (4.41, 4.98)	0.252
ALT, median (P25, P75), U/L	13.00 (10.00, 19.00)	14.00 (10.00, 21.00)	< 0.001
AST, median (P25, P75), U/L	17.00 (14.00, 20.00)	17.00 (14.00, 21.00)	0.044
AIB, median (P25, P75), g/L	41.80 (39.70, 44.00)	41.80 (39.50, 44.00)	0.511
TBil, median (P25, P75), umol/L	8.80 (6.90, 11.30)	8.70 (6.80, 11.40)	0.705
Scr, median (P25, P75), umol/L	46.00 (41.00, 55.00)	46.00 (41.00, 56.00)	0.227
BUN, median (P25, P75), mmol/L	2.70 (2.30, 3.20)	2.77 (2.30, 3.30)	0.118
SBP1	110.00 (102.00, 118.00)	110.50 (102.00, 119.88)	0.066
SBP2	110.33 (103.25, 119.00)	112.00 (104.50, 120.00)	< 0.001
SBP3	113.17 (106.75, 119.67)	114.75 (108.00, 121.24)	< 0.001
DBP1	67.00 (61.00, 72.00)	68.00 (62.00, 73.00)	0.002
DBP2	66.00 (60.67, 71.00)	67.00 (61.54, 72.00)	< 0.001
DBP3	67.80 (63.50, 72.25)	69.12 (64.25, 74.00)	< 0.001
WEIGHT1	54.26 (7.48)	54.67 (7.97)	0.105
WEIGHT2	57.63 (7.53)	58.07 (8.14)	0.081
WEIGHT3	63.39 (7.87)	63.12 (8.46)	0.314
SFH2	21.50 (20.00, 23.00)	21.50 (20.00, 23.00)	0.142
SFH3	30.00 (28.80, 31.25)	29.06 (27.50, 30.40)	< 0.001
MAC2	83.00 (79.00, 87.50)	84.00 (80.00, 88.50)	< 0.001
MAC3	92.00 (88.33, 96.00)	91.50 (87.40, 96.00)	0.019
diffSBP12	0.50 (−5.00, 7.00)	1.00 (−5.00, 8.50)	0.021
diffSBP23	2.17 (−3.33, 7.50)	2.04 (−3.38, 8.00)	0.766
diffSBP13	3.00 (−3.50, 9.40)	4.00 (−3.23, 11.19)	0.005
diffDBP12	−0.50 (−5.00, 4.00)	0.00 (−4.63, 4.00)	0.110
diffDBP23	1.75 (−2.00, 5.67)	2.17 (−2.15, 6.25)	0.156
diffDBP13	1.00 (−3.80, 5.80)	1.45 (−3.00, 7.00)	0.007
diffWEIGHT12	3.25 (2.25, 4.50)	3.25 (2.25, 4.50)	0.625
diffWEIGHT23	5.62 (4.17, 7.17)	4.81 (3.38, 6.33)	< 0.001
diffWEIGHT13	9.00 (7.05, 11.03)	8.04 (6.25, 10.50)	< 0.001
diffSFH23	8.64 (7.17, 10.00)	7.50 (5.75, 9.00)	< 0.001
diffMAC23	8.80 (6.75, 10.90)	7.25 (5.40, 9.33)	< 0.001

Data were median (P25, P75) or mean (SD). SD, standard deviation. For definitions of features see Supplementary Table S1.

^*^Comparing feature distribution between preterm birth and full-term birth is implemented with rank sum test for continuous variables and chi-square test for categorical variables.

FBG, Fasting blood glucose; ALT, Alanine aminotransferase; AST, Aspartate aminotransferase; AIB, Albumin; TBil, Total bilirubin; Scr, Serum creatinine; BUN, Serum urea nitrogen; DBP, diastolic blood pressure; SBP, systolic blood pressure; SFH, symphysis fundal height; MAC, maternal abdominal circumference.

### Maternal measurements during antenatal care visits

The level of systolic blood pressure (SBP) in middle and late pregnancy was higher in the PTB group than in the full-term group (all *P* < 0.001), with higher increases in SBP levels during pregnancy (diffSBP13, 4.0 vs. 3.0, *P* = 0.005) (Table 1). The diastolic blood pressure (DBP) levels at all three gestational intervals were higher in the PTB group than in the full-term group (all *P* < 0.01), with higher increases in DBP levels during pregnancy (diffDBP13, 1.45 vs. 1.00, *P* = 0.007). The increases of symphysis fundal height (SFH) and maternal abdominal circumference (MAC) in late pregnancy were more in pregnant women who delivered full-term birth than those who delivered preterm (all *P* < 0.001) (Table 1). The maternal characteristics of the PTB group and the full-term group in the training set balanced by the hybrid resampling or under-sampling are shown in Supplementary Table S5.

### Feature selection

Supplementary Table S6 presents the model performances of four ML algorithms with all 49 features using the balanced training data set. The highest AUC value was achieved in the CatBoost model developed using the training data set balanced with hybrid resampling (the AUC value: 0.679), and in the Stacked model developed using the training data set balanced with the under-sampling technique (the AUC value: −0.692). Supplementary Tables S7, S8 list the permutation-based feature importance with hybrid resampling or under-sampling. The top five features derived from models using the training data set balanced by hybrid resampling were: SFH3, diffMAC23, diffSFH23, DBP3, and FBG. The top five features derived from models using the training data set balanced by under-sampling were: SFH3, diffSFH23, diffMAC23, neonatal sex, and hemoglobin. The predictors of PTB selected according to the different inclusion rules were combined with five additional features of maternal weight and height at registration, maternal age, neonatal sex, and parity to develop prediction models (Supplementary Table S9).

### Model performance

**Figure 2:**
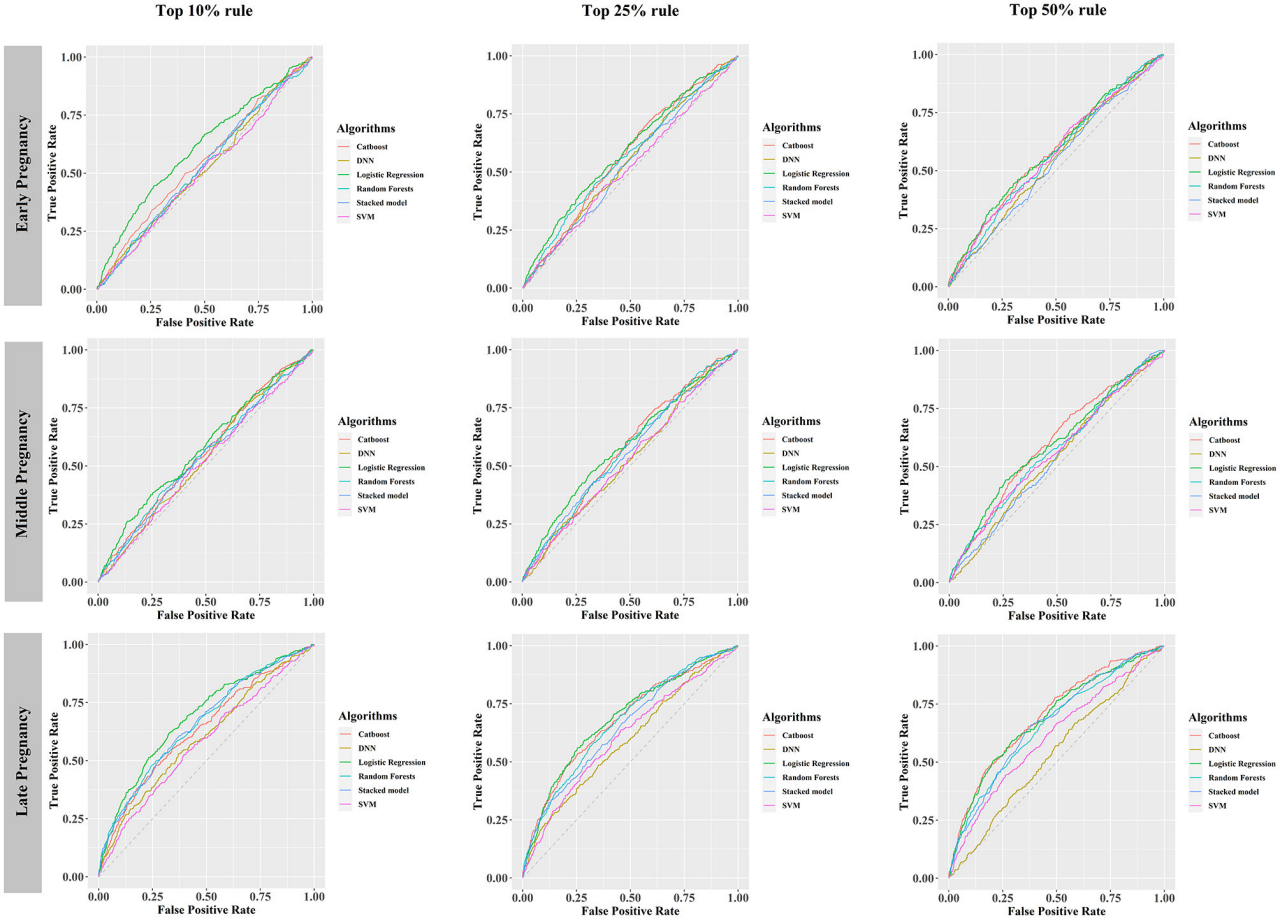
Receiver operating characteristic curves of six prediction models, developed using the training data set balanced by hybrid resampling, validated in the testing data set. DNN, deep neural networks; SVM, support vector machine.

**Figure 3:**
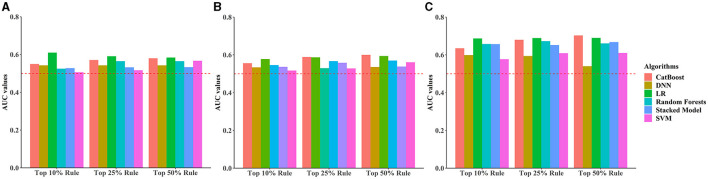
Bar plots comparing area under the receiver operating characteristic curve (AUC) values across all prediction models stratified by different rules for selecting the number of predictors and pregnancy intervals. **(A)** Early pregnancy, **(B)** Middle pregnancy, **(C)** Late pregnancy. The horizontal red dotted line indicates an AUC value of 0.5.

Under different rules (top 10%, top 25%, top 50%) to select the number of included predictors, early, middle, and late pregnancy models were constructed considering the predictors available at different gestational intervals (Table 2, Supplementary Table S10). The ROC curves for all models in the training and validation data sets are illustrated in Figure 2 and Supplementary Figures S3–S5. Among all predictive models, the late pregnancy models performed best, with the highest AUC value achieved by the CatBoost model (the AUC value: 0.703, 95%CI: 0.672, 0.733; Accuracy: 0.811) with predictors selected by the top 50% rule (Figure 3, Table 2). The hyper-parameter settings for the best-fitting CatBoost model are shown in Supplementary Table S11. All models based on early and middle pregnancy predictors performed less well, irrespective of the top rule according to which the predictors were selected. Among the prediction models with AUC values over 0.680, the highest sensitivity was achieved by the LR-based late pregnancy model (Sen: 0.617) with predictors selected by the top 10% rule. The calibration curves for the best-fitting late pregnancy Catboost models using either resampling method are shown in Supplementary Figure S6.

**Table 2:** Validation performance of models by six machine learning algorithms in predicting preterm birth with hybrid resampling.

**Models**	**Top 10% rule**						**Top 25% rule**						**Top 50% rule**					
	**5-CV Acc in training set**	**Testing set**					**5-CV Acc in training set**	**Testing set**					**5-CV Acc in training set**	**Testing set**				
		**AUC (95% CI)**	**Acc**	**Sen**	**Spe**	**Optimal threshold**		**AUC (95% CI)**	**Acc**	**Sen**	**Spe**	**Optimal threshold**		**AUC (95% CI)**	**Acc**	**Sen**	**Spe**	**Optimal threshold**
**Early pregnancy models (**<**18 gestational weeks)**																		
CatBoost	0.984	0.550 (0.516, 0.584)	0.586	0.500	0.590	0.499	0.919	0.571 (0.539, 0.603)	0.425	0.721	0.412	0.451	0.950	0.580 (0.547, 0.614)	0.653	0.473	0.661	0.481
Random Forests	0.997	0.525 (0.492, 0.558)	0.325	0.745	0.306	0.137	1.000	0.565 (0.533, 0.597)	0.769	0.215	0.790	0.204	1.000	0.565 (0.533, 0.597)	0.692	0.402	0.706	0.174
Stacked model	0.997	0.528 (0.495, 0.561)	0.354	0.725	0.337	0.098	1.000	0.532 (0.499, 0.565)	0.543	0.523	0.544	0.150	1.000	0.533 (0.500, 0.565)	0.312	0.775	0.290	0.108
DNN	0.532	0.543 (0.515, 0.572)	0.519	0.560	0.516	0.062	0.647	0.543 (0.510, 0.575)	0.364	0.732	0.347	0.001	0.886	0.543 (0.510, 0.575)	0.364	0.732	0.347	0.001
SVM	0.942	0.506 (0.472, 0.540)	0.486	0.557	0.483	0.088	0.959	0.517 (0.483, 0.552)	0.615	0.440	0.623	0.195	0.800	0.567 (0.533, 0.601)	0.448	0.685	0.438	0.321
LR	0.550	0.610 (0.576, 0.644)	0.722	0.446	0.735	0.461	0.581	0.591 (0.557, 0.625)	0.711	0.416	0.724	0.536	0.577	0.584 (0.550, 0.618)	0.684	0.446	0.695	0.544
**Middle pregnancy models (**<**26 gestational weeks)**																		
CatBoost	0.942	0.556 (0.523, 0.589)	0.288	0.826	0.263	0.417	0.957	0.589 (0.554, 0.623)	0.740	0.423	0.755	0.494	0.972	0.600 (0.567, 0.632)	0.451	0.725	0.438	0.463
Random Forests	0.999	0.546 (0.514, 0.578)	0.700	0.379	0.715	0.155	1.000	0.567 (0.536, 0.599)	0.660	0.430	0.670	0.301	1.000	0.570 (0.538, 0.601)	0.851	0.215	0.880	0.254
Stacked model	0.999	0.537 (0.504, 0.570)	0.667	0.393	0.680	0.221	1.000	0.558 (0.526, 0.590)	0.804	0.255	0.829	0.235	1.000	0.538 (0.506, 0.571)	0.453	0.621	0.446	0.134
DNN	0.532	0.534 (0.501, 0.566)	0.356	0.752	0.337	0.030	0.876	0.530 (0.496, 0.563)	0.278	0.812	0.253	2.519^*^10^−19^	0.781	0.536 (0.503, 0.569)	0.381	0.705	0.366	0.056
SVM	0.904	0.517 (0.484, 0.551)	0.567	0.487	0.570	0.266	0.827	0.528 (0.494, 0.561)	0.457	0.604	0.451	0.273	0.909	0.561 (0.526, 0.596)	0.773	0.322	0.793	0.645
LR	0.560	0.578 (0.544, 0.612)	0.740	0.376	0.757	0.547	0.573	0.587 (0.552, 0.621)	0.673	0.473	0.683	0.540	0.576	0.594 (0.559, 0.628)	0.725	0.443	0.738	0.560
**Late pregnancy models (**<**37 gestational weeks)**																		
CatBoost	0.996	0.635 (0.601, 0.668)	0.724	0.480	0.735	0.438	0.950	0.680 (0.647, 0.713)	0.736	0.537	0.745	0.536	0.955	0.703 (0.672, 0.733)	0.811	0.470	0.827	0.478
Random Forests	1.000	0.658 (0.630, 0.687)	0.903	0.232	0.934	0.310	1.000	0.673 (0.646, 0.701)	0.895	0.238	0.925	0.298	1.000	0.661 (0.633, 0.689)	0.884	0.258	0.912	0.239
Stacked model	1.000	0.657 (0.629, 0.685)	0.936	0.141	0.973	0.457	1.000	0.652 (0.624, 0.681)	0.892	0.252	0.821	0.317	1.000	0.668 (0.640, 0.695)	0.916	0.184	0.950	0.363
DNN	0.772	0.540 (0.507, 0.572)	0.626	0.530	0.630	0.001	0.876	0.594 (0.560, 0.628)	0.722	0.399	0.737	3.946^*^10^−5^	0.992	0.540 (0.507, 0.572)	0.428	0.668	0.417	6.431^*^10^−9^
SVM	0.902	0.577 (0.542, 0.612)	0.601	0.527	0.604	0.306	0.876	0.609 (0.575, 0.643)	0.549	0.638	0.545	0.330	0.901	0.610 (0.576, 0.644)	0.722	0.446	0.735	0.461
LR	0.631	0.687 (0.655, 0.719)	0.673	0.617	0.676	0.550	0.634	0.689 (0.657, 0.721)	0.709	0.594	0.714	0.567	0.640	0.690 (0.658, 0.722)	0.782	0.507	0.795	0.618

AUC, area under the curve; DNN, deep neural networks; LR, logistic regression; SVM, support vector machine; 5-CV, five-fold cross validation.

Each prediction model includes the predictors according to its own rule of feature selection, with additional five features of parity, maternal age and height and weight at register, and the neonatal sex.

The top 10% predictors include SFH3, diffMAC23, diffSFH23, DBP3, and FBG.

The top 25% predictors include SFH3, diffMAC23, diffSFH23, DBP3, FBG, leukocyte, hemoglobin, beats, BUN, and platelet.

The top 50% predictors include SFH3, diffMAC23, diffSFH23, DBP3, FBG, leukocyte, hemoglobin, beats, BUN, platelet, TBil, AIB, length days of a menstrual cycle, number of visiting before 24 gestational weeks, diffSBP13, diffWEIGHT12, diffWEIGHT23, SFH2, AST, diffWEIGHT13, diffSBP23, age at menarche, and diffDBP23.

**Figure 4:**
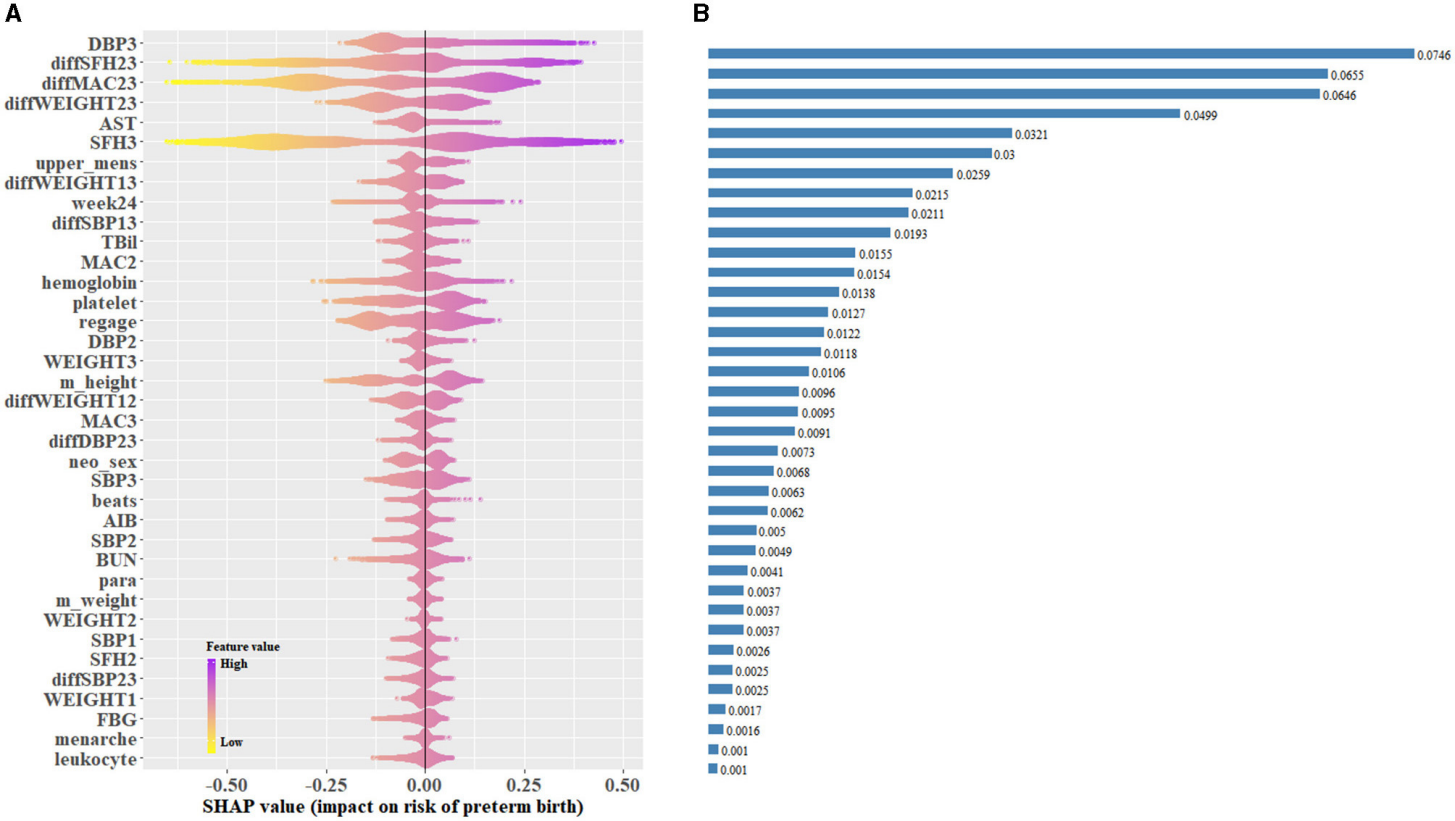
Catboost-based SHAP values of the late pregnancy model using the top 50% predictors in the testing data set. **(A)** SHAP values for individuals in the testing data set were predicted and plotted for each predictor. **(B)** Bar plot of the median absolute SHAP values for each predictor. SHAP, Shapley additive explanations.

SHAP values represent the contribution of each included predictor on an individual's prediction of PTB. SHAP values based on the CatBoost-based late pregnancy model in which predictors were selected by the top 50% rule, with the median absolute SHAP values used to rank feature importance, are shown in Figure 4. The ten highest impact features were DBP3 (median absolute SHAP value: 0.075), diffSFH23 (0.066), diffMAC23 (0.065), diffWEIGHT23 (0.050), AST (0.032), SFH3 (0.030), length days of a menstrual cycle (0.026), diffWEIGHT13 (0.021), number of antenatal visits before 24 weeks (0.019), and diffSBP13 (0.016) (Supplementary Table S12). Among the ten highest impact predictors, the level of DBP in the late pregnancy period (DBP3), AST level at registration, more than 4 antenatal visit before 24 weeks, and increase in SBP during the whole pregnancy (diffSBP13) were associated with increased risk of PTB, whereas higher level in SFH3, diffSFH23, diffMAC23, diffWEIGHT23, and diffWEIGHT13 were associated with decreased risk of PTB (Supplementary Table S13).

## Discussion

This study used ML algorithms to establish prediction models for PTB across three gestational intervals, applying feature selection that synthesized multiple rankings of feature importance derived from the ML models. Our study developed a Catboost-based model with predictors from the routine antenatal care available after 26 completed weeks, achieving an accuracy of over 0.8. Addition of features that are strongly associated with preterm birth, such as previous PTB history, pregnancy hypertension syndromes, gestational diabetes, and ultrasound measurements, including cervical length, into the current best-fitting model, is likely to further improve the model using the CatBoost algorithm and could lead to improved evaluation of the risk of PTB during pregnancy. The high-impact predictors found by our study could feed future, more efficient ML algorithms to achieve better predictive power. In addition, our findings indicate that obstetric doctors should particularly monitor the frequency of antenatal visits before 24 weeks, change in maternal blood pressure, weight, symphysis fundal height and abdominal circumference in late pregnancy.

We used electronic health records to feed the ML models, adding new models for predicting PTB. Compared with previous similar studies, some other prediction models with AUC values > 0.80 outperformed the model we developed (AUC: 0.703), but the study design, the predictors, and the analysis processes used by other studies are more likely to lead to overestimation of model performance and clinical implications (Arabi Belaghi et al., 2021; Speiser, 2021; Sun et al., 2022). Arabi Belaghi et al. (2021) proposed a prediction model during the second trimester which achieved an AUC of 0.80 with artificial neural networks, but their predictors were selected by univariate logistic regression instead of the state-of-the-art ML algorithms, and the predictor maternal complication they used was not defined. Sun et al. developed a prediction model using Random Forest and obtained an AUC value of approximate 0.90, but the non-nested 1:1 case-control study design used could produce great overestimation of the model performance (Sun et al., 2022). Another study added preconception thyroid-stimulating hormone (TSH) levels into prediction models, obtaining the AUC value of 0.812, however, measurement of preconception TSH levels is not included in routine items of preconception examination and therefore prevented its inclusion in our models (Sun et al., 2021).

This study found similar model performance to predict PTB at different pregnancy periods across differing ML algorithms. Stacked Model did not perform better than other individual algorithms. Some important predictors such as gestational diabetes and previous PTB were not included, which possibly resulted in loss of model performance. The AUC value of the CatBoost model was the highest among all ML algorithms. The CatBoost model outperformed the current state-of-the-art implementations of Gradient Boosting Decision Trees to address categorical features without converting them into number, and modify classical gradient boosting algorithms to achieve an unbiased gradient to relieve the overfitting problem (Dorogush et al., 2018). Some researchers reported that CatBoost models achieved outstanding predictive power in gestational diabetes mellitus, suggesting that this algorithm has advantages in the field of neonatal and pregnancy science (Kumar et al., 2022; Zhang and Wang, 2022). Moreover, we found that the predictive power of CatBoost was best after 26 weeks' gestation with the AUC of 0.70, suggesting that there may be room for providing preventative and therapeutic interventions to reduce the risk of PTB after 26 gestational weeks.

We found that the risk of PTB was associated with more than four antenatal care visits before 24 weeks. Given that the frequency of antenatal care visits before delivery may be a confounding influence, we analyzed the frequency of antenatal visits before 24 weeks' gestation - the minimum value of gestational age at birth in our study data set - to assess its relationship with PTB. Compared with 2 to 4 antenatal care visits, singleton pregnancies with > 4 visits before 24 weeks were associated with increased risk of PTB. Notably, singleton pregnancies with > 4 visits of antenatal care before 24 weeks' gestation might be attributable to maternal complications or mental health problems (Nath et al., 2017; Kumar and Dhillon, 2021).

We found that the level of AST at registration was associated with PTB. It has been previously reported that serum AST, a hematological measurement to evaluate liver function, is associated with PTB (Zhuang et al., 2017). The cause of high level of AST during pregnancy may be specific or non-specific liver diseases, indicating a potential risk of abnormal liver function that is highly related to adverse perinatal outcomes (Liu et al., 2022). A retrospective study that investigated the trajectories of AST levels during normal pregnancy found the AST level mostly remained unchanged during pregnancy, and indicated that monitoring of AST levels during pregnancy could help early recognition and diagnosis of impaired liver function (Ushida et al., 2022).

Our study has a number of strengths. We analyzed over 20,000 singleton pregnancies with complete data to develop ML models to predict preterm birth. The large sample from one site allowed us to develop robust predictive models, with less bias due to a consistent procedure of data collection. We report the whole process of data pre-processing (data cleaning, splitting, and resampling) and data engineering to reduce statistical bias and improve predictive power. The use of multiple ML algorithms for feature importance ranking and feature selection is the unique highlight of this study, compared with most studies using a single ML algorithm or univariate correlation to perform feature selection (Koivu and Sairanen, 2020; Arabi Belaghi et al., 2021; Speiser, 2021; Zhao et al., 2021). We are the first to use multiple ML algorithms to conduct feature selection in this field, referring to a principle from ensemble models that an ensemble approach outperforms any individual model (Dietterich, 2000). The feature importance list is not identical across different ML algorithms, so considering multiple ML algorithms to produce the final ranking by averaging multiple feature importance scores reduces potential ranking variance derived from different algorithms. Last but not least, to our knowledge, this is the first study to use the CatBoost algorithm to predict PTB, and the CatBoost algorithm performed overall better than other ML algorithms and logistic regression.

Our study has some limitations. Firstly, the predictive ability of the optimal CatBoost model was modest, with an AUC value of slightly over 0.70, mainly due to the lack of some important predictors such as previous PTB history and gestational diabetes mellitus. Second, the indices collected from maternal blood and urine tests at registration had limited power to predict PTB, apart from AST. Third, we divided the pregnancy period before 37 weeks into three time intervals that limited the deep learning ability of DNN to achieve their maximum predictive performance (Zhang et al., 2022). Fourth, we did not have information on the length of the uterine cervix, which is a known predictor of PTB. Fifth, although we used hybrid and under-sampling methods in the training data set to improve model performance, we did not balance the validation and testing sets to assess model performance, as some previous studies did (Nieto-Del-Amor et al., 2022; Kyparissidis Kokkinidis et al., 2023). Finally, there may have been misclassification and selection bias in our electronic health record-based study. However, preterm birth was defined according to the gestational age determined by the ultrasound scan at the first antenatal visits, thereby limiting the potential for outcome misclassification.

## Conclusion

The CatBoost-based PTB prediction model is a promising predictive tool to help decision making for physicians in clinical practice, including decisions regarding referral to a preterm birth clinic, ultrasound assessment of the cervical length, and administration of preventative interventions, such as progesterone. The number of antenatal care visits before 24 weeks' gestation, AST at registration, symphysis fundal height, maternal weight, abdominal circumference, and blood pressure were identified as strong predictors after 26 completed weeks. The model may be improved and developed further with additional strong predictors.

## Data availability statement

The original contributions presented in the study are included in the article/Supplementary material, further inquiries can be directed to the corresponding authors.

## Ethics statement

The studies involving humans were approved by Ethics Committee of the Second Hospital Affiliated to Wenzhou Medical University. The studies were conducted in accordance with the local legislation and institutional requirements. The ethics committee/institutional review board waived the requirement of written informed consent for participation from the participants or the participants' legal guardians/next of kin because anonymised data was derived from the regional pregnancy surveillance data system.

## Author contributions

Q-YY: Conceptualization, Formal analysis, Funding acquisition, Investigation, Methodology, Resources, Validation, Visualization, Writing—original draft. YL: Data curation, Writing—review & editing. Y-RZ: Data curation, Writing—review & editing. X-JY: Conceptualization, Investigation, Writing—review & editing. JH: Conceptualization, Investigation, Methodology, Resources, Supervision, Writing—review & editing.
