# Supplementary material for: Predicting risk of preterm birth in singleton pregnancies using machine learning algorithms
Source: Front Big Data. 2024 Feb 29;7:1291196. doi: 10.3389/fdata.2024.1291196 (PMC10941650; doi:10.3389/fdata.2024.1291196)
Supplement: Supplementary file 1 [file Data_Sheet_1.PDF]

Supplementary Material

Predicting Risk of Preterm Birth in Singleton Pregnancies Using Machine Learning Algorithms

Qiu-Yan Yu, Ying Lin, Yu-Run Zhou, Xin-Jun Yang, Joris Hemelaar

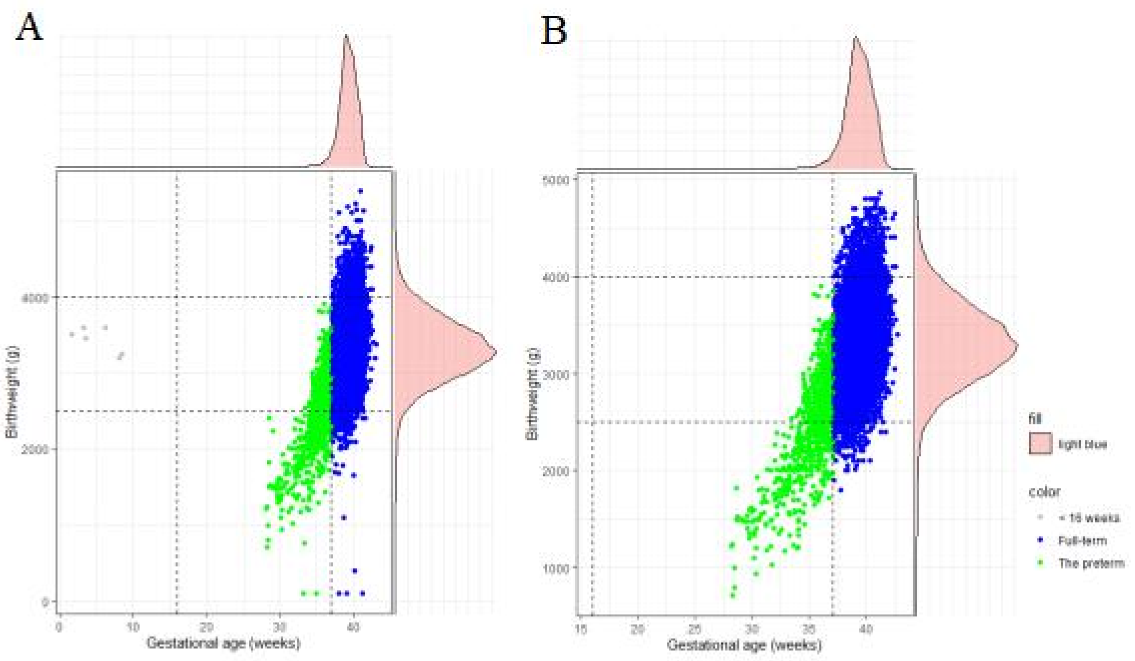

Figure S1. The distribution of the birthweight and gestational age in 22650 pregnancies with the complete data.

(A) cases with < 24 completed weeks' gestation but with birthweight >1000 g, or those cases with > 24 completed weeks' gestation but had weight Z-scores beyond the range of -3 and 3 according to Intergrowth-21st birthweight standards; (B) excluded the cases that stated above.

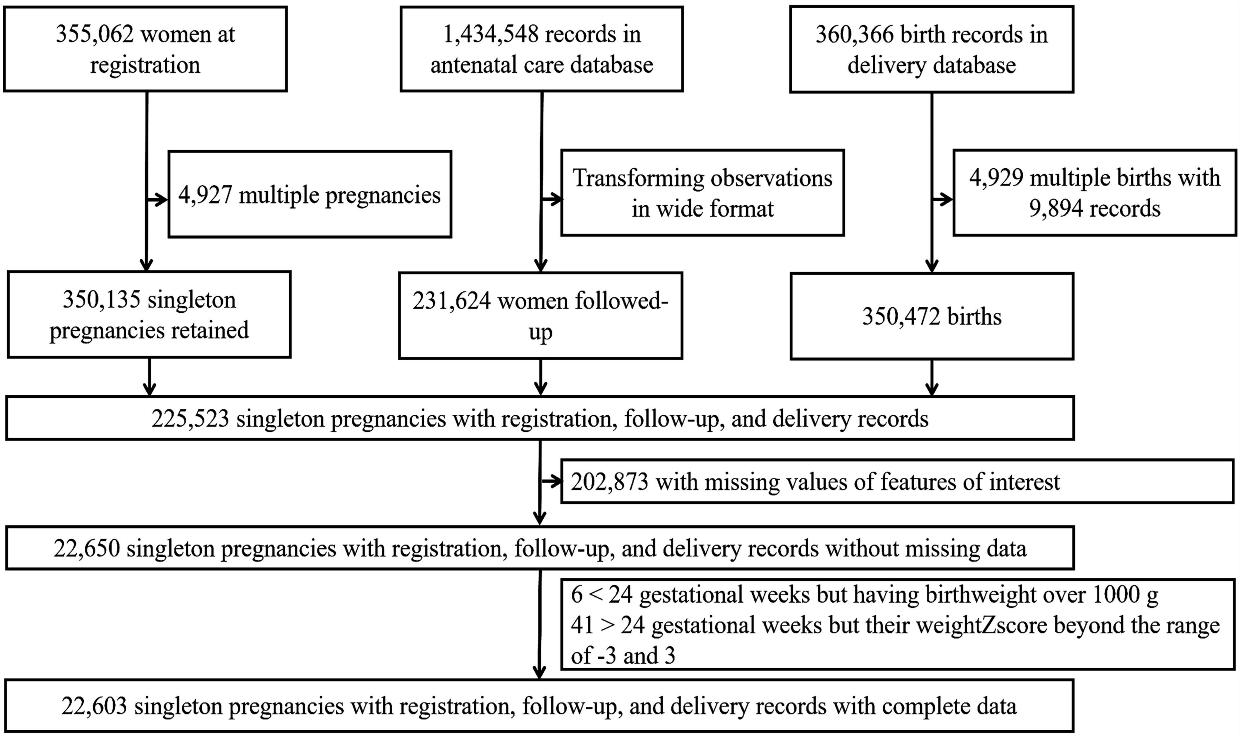

Figure S2. The flowchart to select pregnant women.

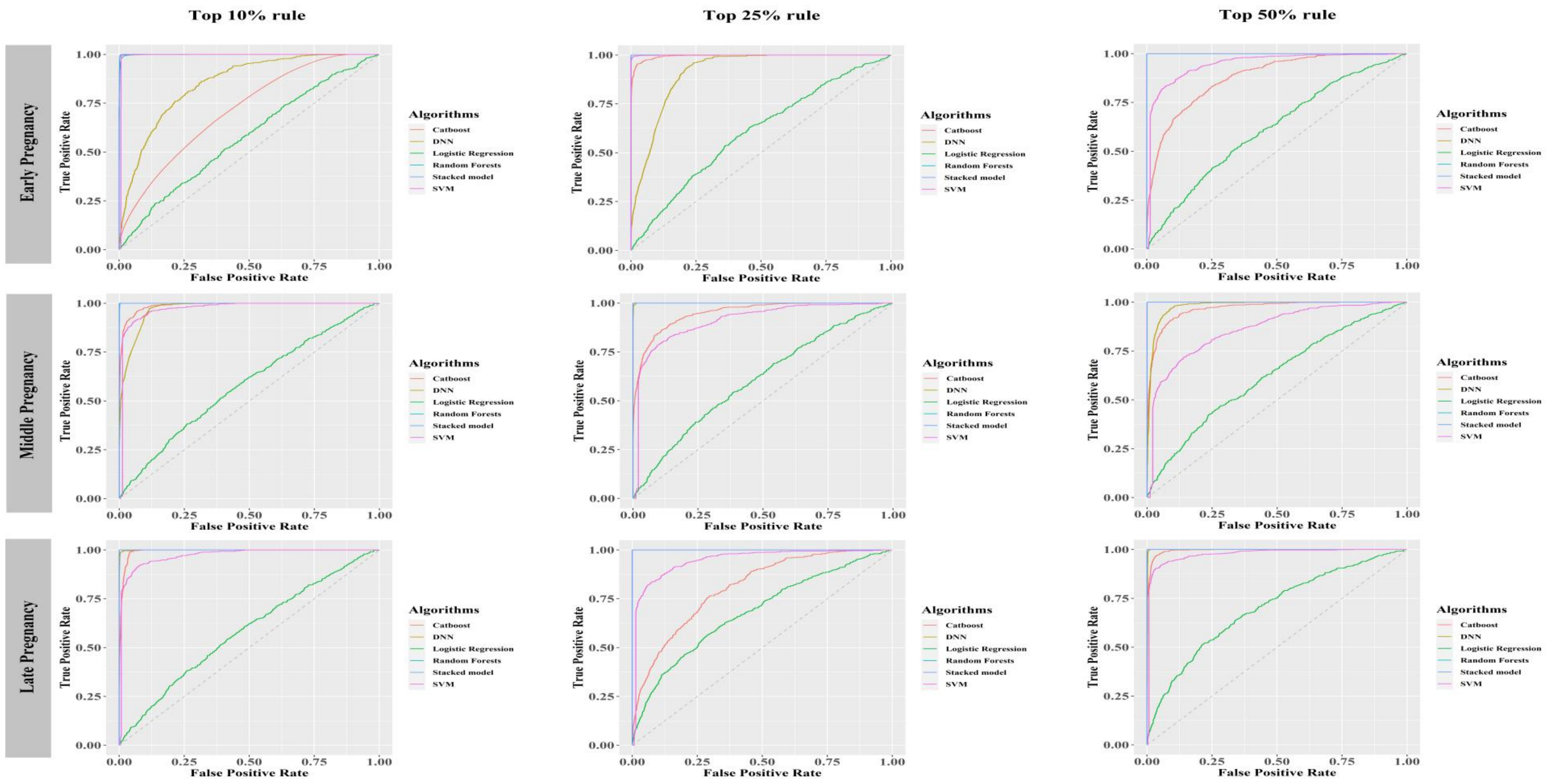

Figure S3. Receiver operating characteristic curves for six prediction models developed using the training set balanced by the hybrid resampling. DNN, deep neural networks; SVM, support vector machine.

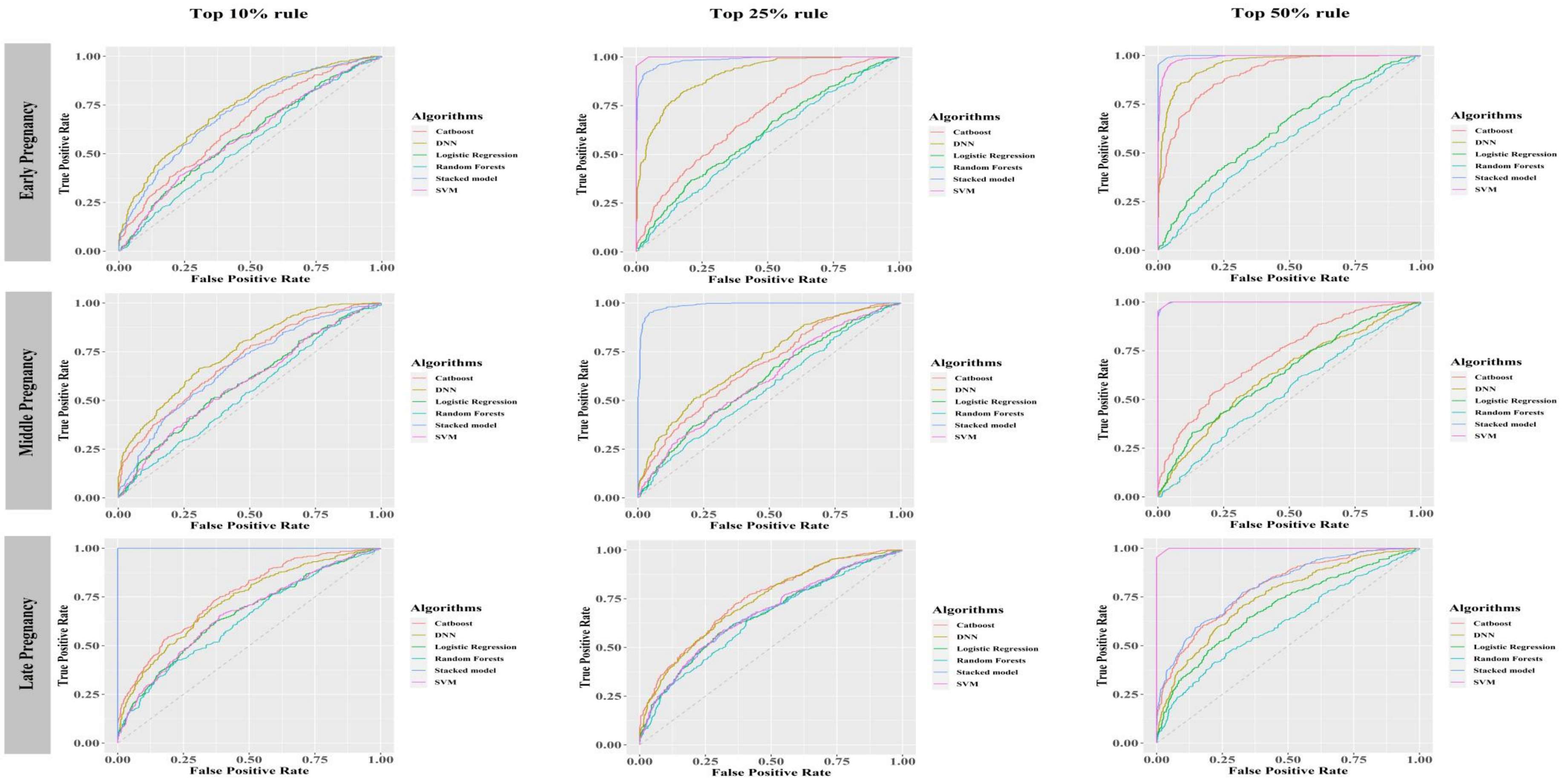

Figure S4. Receiver operating characteristic curves for six prediction models developed using the training set balanced by the under-sampling. DNN, deep neural networks; SVM, support vector machine.

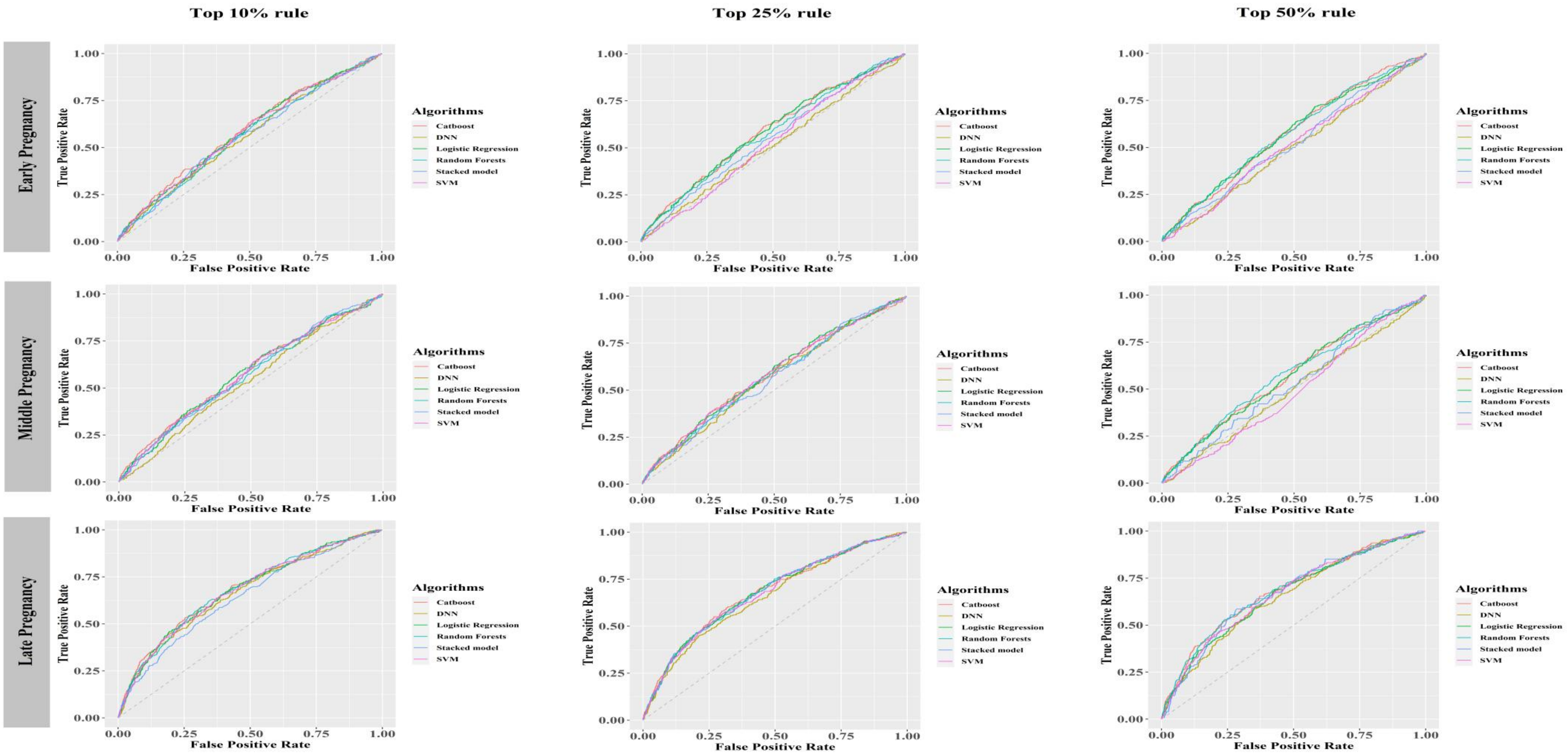

Figure S5. Receiver operating characteristic curves for six prediction models, developed using the training set balanced by the under-sampling, validated in the testing set.

DNN, deep neural networks; SVM, support vector machine.

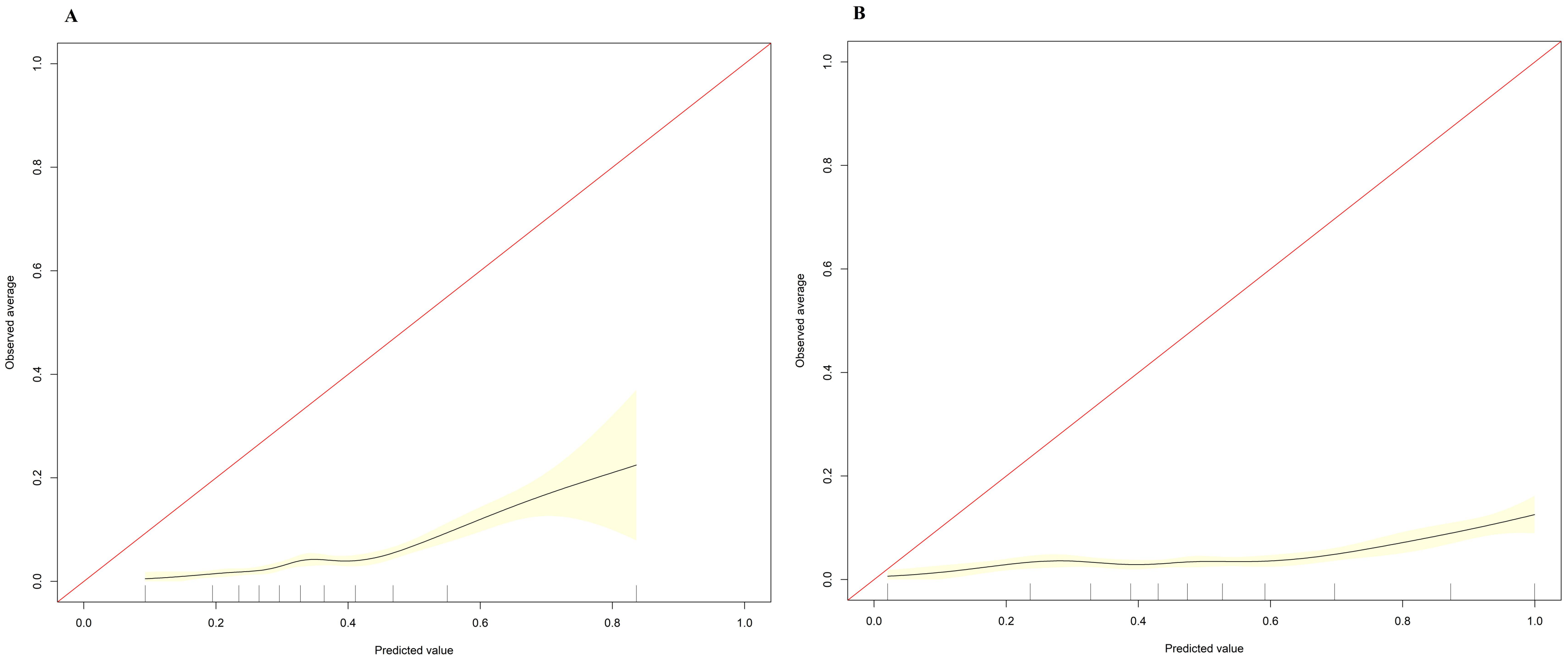

Figure S6. Calibration curves in the testing data set. (A) the best-fitting model, the late pregnancy CatBoost-based model, developed by the training set balanced by the hybrid resampling. (B) the best-fitting model, the late pregnancy Stacked model, developed by the training set balanced by the under-sampling.

Table S1. List of maternal features applied to predict preterm birth.

F.code	F.name	F.type	F.source	F.code	F.name	F.type	F.source
regage	Maternal age, y	Numeric	Registration data	SBP1	SBP1#, mmHg	Numeric	Follow-up data
week24	Antenatal visiting number before 24 weeks	Numeric	Follow-up data	SBP2	SBP2##, mmHg	Numeric	Follow-up data
Firstgw	Ultrasound gestational weeks at 1st visit	Numeric	Follow-up data	SBP3	SBP3###, mmHg	Numeric	Follow-up data
gwage	Unltrasound gestational age, months	Numeric	Delivery records	DBP1	DBP1#, mmHg	Numeric	Follow-up data
m_weight	Maternal weight, kg	Numeric	Registration data	DBP2	DBP2##, mmHg	Numeric	Follow-up data
m_height	Maternal height, cm	Numeric	Registration data	DBP3	DBP3###, mmHg	Numeric	Follow-up data
Menarche	Age at menarche, y	Numeric	Registration data	Weight1	Weight1#, kg	Numeric	Follow-up data
upper_mens	Length of a menstrual cycle, days	Numeric	Registration data	Weight2	Weight2##, kg	Numeric	Follow-up data
Mensday	length of a menstrual period, days	Numeric	Registration data	Weight3	Weight3###, kg	Numeric	Follow-up data
Para	Times of parity	Numeric	Registration data	SFH2	SFH2##, cm	Numeric	Follow-up data
Beats	Maternal heart rate, times per minute	Numeric	Registration data	SFH 3	SFH 3###, cm	Numeric	Follow-up data
Hemoglobin	Hemoglobin, g/L	Numeric	Registration data	MAC2	MAC2##, cm	Numeric	Follow-up data
Leukocyte	White blood cell count, 10^9/L	Numeric	Registration data	MAC3	MAC3###, cm	Numeric	Follow-up data
Platelet	Platelet count, 10^9/L	Numeric	Registration data	Neo_sex	Neonatal sex, male	Binary	Delivery records
FBG	Fasting blood glucose, mmol/L	Numeric	Registration data	diffSBP13	Difference between SBP3 and SBP1	Numeric	-
ALT	Alanine aminotransferase, U/L	Numeric	Registration data	diffSBP12	Difference between SBP2 and SBP1	Numeric	-
AST	Aspartate aminotransferase, U/L	Numeric	Registration data	diffSBP23	Difference between SBP3 and SBP2	Numeric	-
AIB	Albumin, g/L	Numeric	Registration data	diffDBP13	Difference between DBP3 and DBP1	Numeric	-
TBil	Total bilirubin, umol/L	Numeric	Registration data	diffDBP12	Difference between DBP2 and DBP1	Numeric	-
Scr	Serum creatinine, umol/L	Numeric	Registration data	diffDBP23	Difference between DBP3 and DBP2	Numeric	-
BUN	Serum urea nitrogen, mmol/L	Numeric	Registration data	diffWEIGHT13	Difference between WEIGHT3 and WEIGHT1	Numeric	-
Occupation	Occupation	Nominal	Registration data	diffWEIGHT12	Difference between WEIGHT2 and WEIGHT1	Numeric	-
Education	Education	Nominal	Registration data	diffWEIGHT23	Difference between WEIGHT3 and WEIGHT2	Numeric	-
M_complications	Maternal complication, including previa, pregnancy-induced hypertension, or eclampsia during whole pregnancy	Binary	Delivery records	diffSFH23	Difference between SFH3 and SFH2	Numeric	-
m_gysurgery	Gynecologic surgery history	Binary	Registration data	diffMAC23	Difference between MAC3 and MAC2	Numeric	-

F.code, feature code; F.name, feature name; F.type, feature type; F.source, feature resource.

#1st time interval is the period before 18 gestational weeks. ##2th time interval is the period between 18 and 25^{+6} gestational weeks. ###3th time interval is the period between 26 and 36^{+6} gestational weeks.

Table S2. Percentiles of gestational weeks at the first antenatal care visit.

Year	Percentiles, weeks						
	5	10	25	50	75	90	95
2014	11.00	13.00	16.00	18.00	25.00	33.00	36.00
2015	9.00	12.00	14.00	17.00	23.00	32.00	35.00
2016	10.00	12.00	14.00	17.00	22.00	31.00	35.00

Table S3. Hyper-parameter grid search list across five machine learning algorithms for feature selection and prediction modelling.

Models	Set of hyper-parameters
**Random Forest**	# hyperparameter grid<br>hyper_grid.h2o <-list(<br>mtries = floor(n_features * c(.05, .15, .25, .333, .4)),<br>min_rows = c(1, 3, 5, 10),<br>max_depth = c(10, 20, 30),<br>sample_rate = c(.55, .632, .70, .80))<br># random grid search criteria<br>search_criteria <- list(<br>strategy = "RandomDiscrete",<br>stopping_metric = "auc",<br>stopping_tolerance = 0.005,<br>stopping_rounds = 10,<br>max_runtime_secs =30*60)<br>**Optimal hyper-parameters of the full-feature prediction model in the training set with the hybrid resampling:**<br>number_of_trees = 1000; number_of_internal_trees = 1000; model_size_in_bytes = 5429566; min_depth = 17; max_depth = 20; mean_depth = 19.911; min_leaves = 381; max_leaves = 455; mean_leaves =427.68200.<br>**Optimal hyper-parameters of the full-feature prediction model in the training set with the under-resampling:**<br>number_of_trees = 1000; number_of_internal_trees = 1000; model_size_in_bytes = 303933; min_depth = 8; max_depth = 19; mean_depth = 11.138; min_leaves = 38; max_leaves = 50; mean_leaves = 43.62200
**CatBoost**	fit_control=trainControl(method="cv",number=5,classProbs = TRUE)<br>grid1=expand.grid(learning_rate = c(0.1,0.01),<br>depth = c(3,4,6,8,10),<br>iterations=100,<br>l2_leaf_reg = c(1e-3,1,3,5,7,9),<br>rsm = 0.95,<br>border_count = 64)<br>**Optimal hyper-parameters of the full-feature prediction model in the training set with the hybrid resampling:**<br>depth = 10, learning_rate = 0.01, l2_leaf_reg = 7

	**Optimal hyper-parameters of the full-feature prediction model in the training set with the under-resampling:**<br>depth = 3, learning_rate = 0.01, l2_leaf_reg = 7
**Stacked Models**	auto_earlyml <- h2o.automl(x = X, y = Y, training_frame = train_h2o, validation_frame=test_h2o,nfolds = 5,<br>max_runtime_secs = 60 * 120, max_models = 50,include_algos = c("GLM", "DeepLearning",<br>"DRF","XGBoost","GBM","StackedEnsemble"),keep_cross_validation_predictions = TRUE, sort_metric = "AUC", seed =<br>123,stopping_metric = "AUC", stopping_tolerance = 0)<br><br>**Optimal hyper-parameters of the full-feature prediction model in the training set with the hybrid resampling:**<br>GBM model: number_of_trees = 93; number_of_internal_trees = 93; model_size_in_bytes = 410694; min_depth = 11; max_depth =11; mean_depth = 11; min_leaves = 155; max_leaves = 514; mean_leaves = 347.56990.<br>**Optimal hyper-parameters of the full-feature prediction model in the training set with the under-resampling:**<br>GBM model: number_of_trees = 107; number_of_internal_trees = 107; model_size_in_bytes = 503930; min_depth = 11; max_depth =11; mean_depth = 11; min_leaves = 197; max_leaves = 610; mean_leaves = 370.87850.
**SVM**	late.svm.tune=tune(svm,PTB37~.,data=comtrain1,ranges = list(gamma = 2^(-8:1), cost = 2^(0:4)),<br>tunecontrol = tune.control(sampling = "cross",cross=5,best.model=TRUE, performances=TRUE))<br>**Optimal hyper-parameters of the full-feature prediction model in the training set with the hybrid resampling:**<br>gamma = 0.125; cost = 1<br>**Optimal hyper-parameters of the full-feature prediction model in the training set with the under-resampling:**<br>gamma = 0.00390625 ;cost =1
**DNN**	#Hyperparameter tuning<br>def build_model(hp):<br>dnn = Sequential([<br>Flatten(),<br>#providing range for number of neurons in a hidden layer<br>Dense(hp.Int('num_of_neurons1',min_value=16,max_value=128,step=16),activation='relu',<br>input_shape=(X_train.shape[1], 1)),<br>Dense(hp.Int('num_of_neurons2',min_value=16,max_value=128,step=16), activation='relu'),<br>Dense(hp.Int('num_of_neurons3',min_value=16,max_value=128,step=16), activation='relu'),<br>Dense(hp.Int('num_of_neurons4',min_value=16,max_value=128,step=16), activation='relu'),<br>Dropout(hp.Choice('dropout',values=[0.1, 0.2, 0.3])),

	#output layer<br>Dense(1, activation='sigmoid')])<br>#compiling the model<br>dnn.compile(loss='binary_crossentropy',<br>optimizer=keras.optimizers.Adam(hp.Choice('learning_rate',values=[1e-2, 1e-3, 1e-4])), metrics=['accuracy'])<br>return dnn

SVM, support vector machine. Set of hyper-parameters for DNN models are shown in Python code format, and the rest models are shown in R code format.

Table S4. Maternal measurement during the antenatal care visits in the complete data set.

Features	Overall	Full-term birth	Preterm birth	P values*
No.	22603	21657	946	
SBP1	110.00 (102.00, 118.33)	110.00 (102.00, 118.00)	110.50 (102.00, 119.88)	0.066
SBP2	110.50 (103.50, 119.00)	110.33 (103.25, 119.00)	112.00 (104.50, 120.00)	<0.001
SBP3	113.20 (106.80, 119.75)	113.17 (106.75, 119.67)	114.75 (108.00, 121.24)	<0.001
DBP1	67.00 (61.00, 72.00)	67.00 (61.00, 72.00)	68.00 (62.00, 73.00)	0.002
DBP2	66.00 (61.00, 71.00)	66.00 (60.67, 71.00)	67.00 (61.54, 72.00)	<0.001
DBP3	67.83 (63.50, 72.33)	67.80 (63.50, 72.25)	69.12 (64.25, 74.00)	<0.001
WEIGHT1	54.28 (7.50)	54.26 (7.48)	54.67 (7.97)	0.105
WEIGHT2	57.65 (7.56)	57.63 (7.53)	58.07 (8.14)	0.081
WEIGHT3	63.37 (7.89)	63.39 (7.87)	63.12 (8.46)	0.314
SFH2	21.50 (20.00, 23.00)	21.50 (20.00, 23.00)	21.50 (20.00, 23.00)	0.142
SFH3	30.00 (28.75, 31.25)	30.00 (28.80, 31.25)	29.06 (27.50, 30.40)	<0.001
MAC2	83.00 (79.00, 87.50)	83.00 (79.00, 87.50)	84.00 (80.00, 88.50)	<0.001
MAC3	92.00 (88.33, 96.00)	92.00 (88.33, 96.00)	91.50 (87.40, 96.00)	0.019
diffSBP12	0.50 (-5.00, 7.00)	0.50 (-5.00, 7.00)	1.00 (-5.00, 8.50)	0.021
diffSBP23	2.14 (-3.33, 7.50)	2.17 (-3.33, 7.50)	2.04 (-3.38, 8.00)	0.766
diffSBP13	3.00 (-3.50, 9.50)	3.00 (-3.50, 9.40)	4.00 (-3.23, 11.19)	0.005
diffDBP12	-0.50 (-5.00, 4.00)	-0.50 (-5.00, 4.00)	0.00 (-4.63, 4.00)	0.110
diffDBP23	1.78 (-2.00, 5.70)	1.75 (-2.00, 5.67)	2.17 (-2.15, 6.25)	0.156
diffDBP13	1.00 (-3.75, 5.83)	1.00 (-3.80, 5.80)	1.45 (-3.00, 7.00)	0.007
diffWEIGHT12	3.25 (2.25, 4.50)	3.25 (2.25, 4.50)	3.25 (2.25, 4.50)	0.625
diffWEIGHT23	5.60 (4.12, 7.12)	5.62 (4.17, 7.17)	4.81 (3.38, 6.33)	<0.001
diffWEIGHT13	9.00 (7.00, 11.00)	9.00 (7.05, 11.03)	8.04 (6.25, 10.50)	<0.001
diffSFH23	8.60 (7.00, 10.00)	8.64 (7.17, 10.00)	7.50 (5.75, 9.00)	<0.001
diffMAC23	8.75 (6.67, 10.83)	8.80 (6.75, 10.90)	7.25 (5.40, 9.33)	<0.001

Data were median (P25, P75) or mean (SD). SD, standard deviation.

* Comparing feature distribution between preterm birth and full-term birth is implemented with rank sum test for non-normal continuous variables, and *t* test for normal continuous variables.

Table S5. Maternal features in the training set with the hybrid resampling or under-sampling.

Feature	Training set with the hybrid resampling			Training set with the under-resampling		
	Full-term birth	Preterm birth	P values	Full-term birth	Preterm birth	P values
No.	6,148	6,052		520	520	
Maternal age	26.72 (4.49)	27.63 (4.80)	<0.001	26.87 (4.73)	27.72 (4.88)	0.004
Age at menarche	14.06 (1.17)	14.06 (1.28)	0.977	14.03 (1.24)	14.07 (1.27)	0.571
Length days of a menstrual cycle	29.48 (2.65)	29.22 (2.73)	<0.001	29.35 (2.60)	29.27 (2.74)	0.634
Days for a menstrual period	5.21 (1.36)	5.14 (1.38)	0.003	5.25 (1.32)	5.13 (1.37)	0.139
Parity	0.45 (0.53)	0.51 (0.56)	<0.001	0.47 (0.54)	0.52 (0.56)	0.144
Maternal height	159.49 (4.87)	158.72 (4.91)	<0.001	159.38 (4.81)	158.73 (4.93)	0.032
Maternal weight	53.13 (7.26)	53.46 (7.88)	0.016	53.84 (8.04)	53.50 (7.96)	0.502
Maternal heart rate	80.28 (8.99)	80.75 (9.82)	0.006	80.53 (9.15)	80.75 (9.74)	0.699
hemoglobin	124.14 (9.75)	125.70 (9.72)	<0.001	124.67 (9.84)	125.69 (9.66)	0.093
leukocyte	8.13 (1.88)	8.32 (1.94)	<0.001	8.26 (1.81)	8.29 (1.93)	0.810
platelet	217.37 (46.42)	225.39 (47.14)	<0.001	221.35 (51.85)	224.43 (47.17)	0.317
FBG	4.69 (0.45)	4.72 (0.45)	0.007	4.68 (0.45)	4.72 (0.45)	0.163
ALT	16.13 (10.10)	17.71 (11.62)	<0.001	17.75 (11.40)	17.88 (11.64)	0.856
AST	18.05 (6.03)	18.83 (6.83)	<0.001	18.55 (6.58)	18.91 (6.97)	0.391
AIB	41.93 (3.26)	42.02 (3.34)	0.120	41.98 (3.39)	42.07 (3.39)	0.668
TBil	9.53 (3.73)	9.55 (3.86)	0.837	9.59 (3.64)	9.55 (3.86)	0.880
Scr	49.27 (12.56)	49.77 (12.64)	0.029	50.86 (13.71)	49.65 (12.58)	0.138
BUN	2.79 (0.76)	2.85 (0.81)	<0.001	2.82 (0.78)	2.85 (0.81)	0.535
SBP1	110.42 (11.26)	111.12 (12.12)	0.001	110.90 (12.25)	111.04 (12.11)	0.854
SBP2	111.13 (10.70)	112.58 (11.55)	<0.001	112.06 (11.62)	112.58 (11.58)	0.469
SBP3	113.21 (9.25)	114.98 (11.01)	<0.001	113.96 (10.21)	114.90 (10.96)	0.152
DBP1	67.25 (7.94)	68.32 (8.50)	<0.001	67.44 (8.85)	68.22 (8.45)	0.147
DBP2	66.42 (7.48)	67.89 (8.63)	<0.001	66.66 (7.83)	67.71 (8.55)	0.040
DBP3	68.21 (6.54)	70.01 (8.34)	<0.001	68.54 (7.40)	69.84 (8.27)	0.008
WEIGHT1	54.24 (7.48)	54.54 (8.06)	0.035	55.04 (8.39)	54.55 (8.15)	0.336

WEIGHT2	57.60 (7.56)	58.00 (8.29)	0.005	58.35 (8.29)	57.98 (8.39)	0.478
WEIGHT3	63.42 (7.90)	63.13 (8.62)	0.053	64.09 (9.02)	63.11 (8.70)	0.074
SFH2	21.34 (2.28)	21.55 (2.45)	<0.001	21.41 (2.16)	21.58 (2.47)	0.236
SFH3	30.02 (2.24)	29.04 (2.42)	<0.001	29.97 (2.26)	29.00 (2.38)	<0.001
MAC2	83.42 (6.25)	84.41 (6.77)	<0.001	84.12 (6.53)	84.51 (6.84)	0.354
MAC3	92.28 (5.94)	91.92 (6.41)	0.001	92.59 (6.24)	91.92 (6.48)	0.090
diffSBP12	0.00 [-5.50, 7.00]	0.50 [-5.00, 8.00]	0.01	0.75 [-5.27, 8.00]	0.50 [-5.00, 8.00]	0.767
diffSBP23	2.03 [-3.50, 7.50]	2.50 [-3.50, 8.00]	0.085	1.67 [-3.50, 7.00]	2.37 [-3.35, 8.00]	0.257
diffSBP13	2.70 [-4.00, 9.40]	3.50 [-3.33, 11.25]	<0.001	2.63 [-3.80, 9.68]	3.45 [-3.35, 11.50]	0.277
diffDBP12	-0.50 [-5.50, 3.50]	-0.33 [-4.50, 4.50]	<0.001	-0.50 [-6.00, 4.00]	-0.42 [-5.00, 4.00]	0.445
diffDBP23	1.83 [-2.17, 5.67]	2.21 [-2.33, 6.25]	0.016	1.79 [-1.70, 5.71]	2.05 [-2.33, 6.25]	0.582
diffDBP13	0.83 [-3.75, 5.50]	1.33 [-3.00, 6.75]	<0.001	1.00 [-4.00, 6.30]	1.00 [-3.27, 6.69]	0.395
diffWEIGHT12	3.25 [2.25, 4.50]	3.33 [2.25, 4.50]	0.098	3.50 [2.00, 4.50]	3.30 [2.25, 4.50]	0.729
diffWEIGHT23	5.67 [4.30, 7.20]	4.75 [3.50, 6.33]	<0.001	5.48 [4.11, 7.14]	4.78 [3.50, 6.33]	<0.001
diffWEIGHT13	9.00 [7.11, 11.12]	8.00 [6.25, 10.57]	<0.001	8.89 [6.84, 11.00]	8.00 [6.25, 10.50]	0.002
diffSFH23	8.67 [7.17, 10.10]	7.50 [5.67, 9.17]	<0.001	8.50 [6.89, 10.12]	7.50 [5.50, 9.03]	<0.001
diffMAC23	8.83 [6.75, 10.83]	7.25 [5.50, 9.33]	<0.001	8.50 [6.42, 10.80]	7.20 [5.33, 9.27]	<0.001
Number of antenatal visits before 24 weeks(median [IQR])	2.00 [2.00, 3.00]	2.00 [2.00, 3.00]	<0.001	2.00 [2.00, 3.00]	2.00 [2.00, 3.00]	0.007
Maternal complications (%)			<0.001			0.022
No	6129 (99.7)	5917 (97.8)		518 (99.6)	509 (97.9)	
Yes	19 (0.3)	135 (2.2)		2 (0.4)	11 (2.1)	
Gestational weeks at register	16.00 [13.00, 17.00]	16.00 [13.00, 17.00]	<0.001	16.00 [13.00, 17.00]	16.00 [13.00, 17.00]	0.051
Occupation			0.004			0.199

Farmer or fishermen	2346 (38.2)	2373 (39.2)		180 (34.6)	206 (39.6)	
Employee	754 (12.3)	720 (11.9)		77 (14.8)	63 (12.1)	
Business and service industry	733 (11.9)	736 (12.2)		58 (11.2)	61 (11.7)	
Households	1602 (26.1)	1424 (23.5)		148 (28.5)	124 (23.8)	
Others	713 (11.6)	799 (13.2)		57 (11.0)	66 (12.7)	
Education			<0.001			0.987
Primary school and below	275 (4.5)	350 (5.8)		30 (5.8)	31 (6.0)	
Secondary school and high school	3830 (62.3)	3545 (58.6)		310 (59.6)	308 (59.2)	
College and above	2043 (33.2)	2157 (35.6)		180 (34.6)	181 (34.8)	
Gynecological history			<0.001			0.006
Yes	234 (3.8)	443 (7.3)		18 (3.5)	39 (7.5)	
No	5914 (96.2)	5609 (92.7)		502 (96.5)	481 (92.5)	
Neonatal sex			<0.001			0.070
Female	2892 (47.0)	2559 (42.3)		246 (47.3)	216 (41.5)	
Male	3256 (53.0)	3493 (57.7)		274 (52.7)	304 (58.5)	

Data were median [P25, P75], mean (SD), or n (%). SD, standard deviation.

Table S6. Model performance of four ML algorithms with all features developed in the resampled training set and the validation set.

Models	5-fold CV Acc in the training data set	AUC in the testing data set
Full-feature prediction modeling in the training set resampled by the hybrid technique		
Random forests	0·997	0·673
Stacked model	1.000	0·629
Catboost	0·954	0·679
SVM	1.000	0·644
Full-feature prediction modeling in the training set resampled by the KNN-based under-sampling technique		
Random forests	0·522	0·678
Stacked model	1.000	0·692
Catboost	0·557	0·662
SVM	0·734	0·681

Acc,accuracy; AUC, Area under the Receiver Operating Characteristic curve; CV, cross validation; SVM, Support Vector Machine; KNN,K-nearest neighbors.

Table S7. Permutation-based feature importance in the training set resampled by the hybrid resampling.

Predictors	Random Forest		CatBoost		Stacked model		SVM		Average	Final
	Importance	Rank	Importance	Rank	Importance	Rank	Importance	Rank	Importance score	Rank
SFH3	0.0070104	1	0.00005870	1	0.00149536	1	0.00000620	6	2.25	1
diffMAC23	0.0006183	2	0.00000005	3	0.00000024	4	0.00000651	5	3.5	2
diffSFH23	0.0003981	3	0.00000010	2	0.00002330	2	0.00000171	11	4.5	3
DBP3	0.0000575	4	0.00000000	26.5	0.00000208	3	0.00000009	35	17.125	4
FBG	0.0000047	6	0.00000000	26.5	0.00000000	27	0.00000274	10	17.375	5
leukocyte	0.0000129	5	0.00000000	26.5	0.00000000	27	0.00000143	15	18.375	6
hemoglobin	0.0000019	7	0.00000000	26.5	0.00000000	27	0.00000138	16	19.125	7
m_height	0.0000005	8	0.00000000	26.5	0.00000000	27	0.00000137	17	19.625	8
beats	0.0000001	19	0.00000000	26.5	0.00000000	27	0.00000304	9	20.375	9
BUN	0.0000001	13	0.00000000	26.5	0.00000000	27	0.00000121	18	21.125	10
regage	0.0000002	11	0.00000000	26.5	0.00000000	27	0.00000070	21	21.375	11
platelet	0.0000002	10	0.00000000	26.5	0.00000000	27	0.00000054	23	21.625	12
TBil	0.0000001	15	0.00000000	26.5	0.00000000	27	0.00000119	19	21.875	13
AIB	0.0000002	9	0.00000000	26.5	0.00000000	27	0.00000044	25	21.875	14
upper_mens	0.0000000	34.5	0.00000000	26.5	0.00000000	27	0.00002050	1	22.25	15
week24	0.0000000	34.5	0.00000000	26.5	0.00000000	27	0.00001370	2	22.5	16
diffSBP13	0.0000001	17	0.00000000	26.5	0.00000000	27	0.00000107	20	22.625	17
diffWEIGHT12	0.0000000	34.5	0.00000000	26.5	0.00000000	27	0.00000970	3	22.75	18
diffWEIGHT23	0.0000000	34.5	0.00000000	26.5	0.00000000	27	0.00000894	4	23	19
SFH2	0.0000001	18	0.00000000	26.5	0.00000000	27	0.00000057	22	23.375	20
AST	0.0000000	34.5	0.00000000	26.5	0.00000000	27	0.00000391	7	23.75	21
diffWEIGHT13	0.0000000	34.5	0.00000000	26.5	0.00000000	27	0.00000356	8	24	22
diffSBP23	0.0000000	34.5	0.00000000	26.5	0.00000000	27	0.00000170	12	25	23
menarche	0.0000000	34.5	0.00000000	26.5	0.00000000	27	0.00000163	13	25.25	24
diffDBP23	0.0000001	16	0.00000000	26.5	0.00000000	27	0.00000017	32	25.375	25
Scr	0.0000000	34.5	0.00000000	26.5	0.00000000	27	0.00000147	14	25.5	26
DBP2	0.0000001	14	0.00000000	26.5	0.00000000	27	0.00000005	38	26.375	27
MAC2	0.0000001	12	0.00000000	26.5	0.00000000	27	0.00000000	44.5	27.5	28
mensday	0.0000000	34.5	0.00000000	26.5	0.00000000	27	0.00000054	24	28	29
para	0.0000000	34.5	0.00000000	26.5	0.00000000	27	0.00000038	26	28.5	30
ALT	0.0000000	34.5	0.00000000	26.5	0.00000000	27	0.00000036	27	28.75	31
diffSBP12	0.0000000	34.5	0.00000000	26.5	0.00000000	27	0.00000032	28	29	32
diffDBP13	0.0000000	34.5	0.00000000	26.5	0.00000000	27	0.00000029	29	29.25	33
diffDBP12	0.0000000	34.5	0.00000000	26.5	0.00000000	27	0.00000025	30	29.5	34
SBP3	0.0000000	34.5	0.00000000	26.5	0.00000000	27	0.00000022	31	29.75	35
SBP2	0.0000000	34.5	0.00000000	26.5	0.00000000	27	0.00000015	33	30.25	36
DBP1	0.0000000	34.5	0.00000000	26.5	0.00000000	27	0.00000009	34	30.5	37
firstgw	0.0000000	34.5	0.00000000	26.5	0.00000000	27	0.00000009	36	31	38
SBP1	0.0000000	34.5	0.00000000	26.5	0.00000000	27	0.00000006	37	31.25	39
MAC3	0.0000000	34.5	0.00000000	26.5	0.00000000	27	0.00000001	39	31.75	40
WEIGHT3	0.0000000	34.5	0.00000000	26.5	0.00000000	27	0.00000000	44.5	33.125	45
m_gysurgery	0.0000000	34.5	0.00000000	26.5	0.00000000	27	0.00000000	44.5	33.125	45
WEIGHT1	0.0000000	34.5	0.00000000	26.5	0.00000000	27	0.00000000	44.5	33.125	45
m_complications	0.0000000	34.5	0.00000000	26.5	0.00000000	27	0.00000000	44.5	33.125	45
m_weight	0.0000000	34.5	0.00000000	26.5	0.00000000	27	0.00000000	44.5	33.125	45
neo_sex	0.0000000	34.5	0.00000000	26.5	0.00000000	27	0.00000000	44.5	33.125	45
WEIGHT2	0.0000000	34.5	0.00000000	26.5	0.00000000	27	0.00000000	44.5	33.125	45
occupation	0.0000000	34.5	0.00000000	26.5	0.00000000	27	0.00000000	44.5	33.125	45
education	0.0000000	34.5	0.00000000	26.5	0.00000000	27	0.00000000	44.5	33.125	45

Table S8. Permutation-based feature importance in the training set resampled by the under-sampling.

Variable	Random Forest		CatBoost		Stacked model		Average importance score	Final Rank
	Importance	Rank	Importance	Rank	Importance	Rank		
SFH3	0.0206342	2	0.0300575	2	0.0246413	1	1.5	1
diffSFH23	0.0294397	1	0.0287269	3	0.0065939	5	2.75	2
diffMAC23	0.0188924	3	0.0309941	1	0.0084782	4	3	3
neo_sex	0.0096820	9	0.0039697	6	0.0116235	3	5.75	4
hemoglobin	0.0098465	8	0.0023580	9	0.0144952	2	6.25	5
Scr	0.0091845	10	0.0037648	7	0.0021376	12	10.75	6
regage	0.0084246	12	0.0044619	5	0.0015514	16	11.5	7
mensday	0.0059061	22	0.0011864	14	0.0041383	8	11.75	8
m_height	0.0187241	4	0.0075411	4	0.0002792	40	14	9
platelet	0.0110374	5	0.0000000	41	0.0064867	6	15.25	10
MAC2	0.0077496	16	0.0008506	21	0.0018510	14	17.25	11
FBG	0.0107322	6	0.0001930	38	0.0015477	17	17.75	12
occupation	0.0088092	11	0.0006433	26	0.0016198	15	17.75	13
TBil	0.0104179	7	0.0012838	13	0.0007138	28	18.5	14
diffWEIGHT13	0.0078569	14	0.0014196	12	0.0021080	13	18.75	15
BUN	0.0055418	26	0.0014675	11	0.0008173	22	19.75	16
menarche	0.0042567	34	0.0003637	31	0.0032175	9	21.25	17
leukocyte	0.0057822	23	0.0000368	40	0.0024926	11	22.25	18
beats	0.0054789	27	0.0015974	10	0.0007378	26	23	19
education	0.0017437	45	0.0007250	25	0.0042604	7	23.25	20
AIB	0.0061871	21	0.0006036	27	0.0011132	20	23.75	21
m_gysurgery	0.0010244	48	0.0010880	19	0.0030067	10	24.75	22
diffDBP23	0.0067400	18	0.0010947	18	0.0006102	31	26.25	23
diffWEIGHT23	0.0083691	13	0.0009068	20	0.0007840	24	26.5	24
diffSBP13	0.0069194	17	0.0000000	45	0.0007452	25	27	25
diffDBP12	0.0063536	20	0.0002038	37	0.0008321	21	27	26
SBP3	0.0064497	19	0.0011771	15	0.0003347	37	27.75	27
WEIGHT2	0.0057711	24	0.0032757	8	0.0000906	44	29.25	28
SFH2	0.0078143	15	0.0004148	29	0.0001165	43	29.75	29
diffDBP13	0.0048798	30	0.0002899	34	0.0003254	38	29.75	30
DBP3	0.0053717	28	0.0011522	17	0.0003754	35	30.5	31
ALT	0.0056990	25	0.0000000	42	0.0003587	36	31.75	32
AST	0.0039090	40	0.0011760	16	0.0000499	48	31.75	33
m_weight	0.0040551	38	0.0007430	24	0.0008044	23	32.5	34
WEIGHT1	0.0048465	32	0.0003637	32	0.0004734	33	32.75	35
firstgw	0.0033950	41	0.0008138	23	0.0011391	19	32.75	36
diffWEIGHT12	0.0019231	44	0.0000000	46	0.0005233	32	33.5	37
week24	0.0011890	46	0.0000000	47	0.0004031	34	33.5	38
DBP1	0.0050222	29	0.0002947	33	0.0007101	29	34.25	39
WEIGHT3	0.0041624	35	0.0000000	43	0.0011465	18	34.75	40
diffSBP23	0.0039996	39	0.0000398	39	0.0007267	27	35.5	41
SBP1	0.0047578	33	0.0004834	28	0.0000129	49	35.75	42
MAC3	0.0048706	31	0.0002779	35	0.0002977	39	36	43
upper_mens	0.0021967	43	0.0003658	30	0.0000647	46	36	44
para	0.0011409	47	0.0008227	22	0.0002016	41	36.25	45
SBP2	0.0041235	36	-0.0000233	49	0.0006342	30	36.5	46
diffSBP12	0.0040643	37	0.0000000	44	0.0000536	47	39	47
DBP2	0.0033876	42	0.0002047	36	0.0001886	42	41	48
m_complications	0.0000000	49	0.0000000	48	0.0000758	45	47.25	49

Table S9. Predictors for predicting preterm birth for the top 10% rule, the top 25% rule, and the top 50% rule.

Predictors	Top 10% rule	Top 25% rule	Top 50% rule
**Training set with the hybrid resampling**			
**Predictors available at early pregnancy (< 18 gestational weeks)**	**6 predictors:** Parity#, maternal age at register#, maternal weight at register#, maternal height at register#, neonatal sex#, FBG at register.	**11 predictors:** Parity#, maternal weight at register#, Neonatal sex#, maternal age at register, maternal height at register, FBG at register, Leukocyte at register, Hemoglobin at register, Maternal heart rate at register, BUN at register, Platelet at register	**18 predictors:** Parity#, maternal weight at register#, neonatal sex#, maternal age at register, maternal height at register, FBG at register, Leukocyte at register, Hemoglobin at register, Maternal heart rate at register, BUN at register, Platelet at register, TBil at register, AIB at register, Length days of a menstrual cycle, AST at register, Age at menarche, WEIGHT1*, SBP1*
**Predictors available at middle pregnancy (18 to 25^{+6} gestational weeks)**	**2 predictors:** MAC2*, SFH2*	**2 predictors:** MAC2*, SFH2*	**7 predictors:** SFH2, diffWEIGHT12, number of antenatal visiting before 24 weeks, WEIGHT2*, MAC2*, SBP2*, DBP2*
**Predictors available at late pregnancy (26 to 37 gestational weeks)**	**5 predictors:** DBP3, SFH3, diffSFH23, diffMAC23, MAC3*	**5 predictors:** DBP3, SFH3, diffSFH23, diffMAC23, MAC3*	**12 predictors:** MAC3*, WEIGHT3*, SBP3*, SFH3, DBP3, diffSBP13, diffWEIGHT23, diffWEIGHT13, diffSBP23, diffDBP23, diffMAC23, diffSFH23
**Training set with the under-sampling**			
**Predictors available at early pregnancy (< 18 gestational weeks)**	**6 predictors:** Neonatal sex, Hemoglobin at register, Parity#, Maternal age at register#, Maternal height at register#, Maternal	**10 predictors:** Neonatal sex, Scr at register, maternal age at register, days of a menstrual period, maternal height at register, FBG at register, platelet at	**21 predictors:** Neonatal sex, Scr at register, maternal age at register, days of a menstrual period, maternal height at register, FBG at register, platelet at register, hemoglobin at register,occupation, TBil at register, BUN at register, age at menarche, leukocyte at

	weight at register#	register, hemoglobin at register, maternal weight at register#, parity#	register, maternal heart rate at register, maternal education, AIB at register, maternal gynecological history, SBP1*, WEIGHT1*, maternal weight at register#, parity#
**Predictors available at middle pregnancy (18 to 25+6 gestational weeks)**	**2 predictors:** MAC2*, SFH2*	**2 predictors:** MAC2, SFH2*	**4 predictors:** MAC2, SFH2*, WEIGHT2*, DBP2*
**Predictors available at late pregnancy (26 to 37 gestational weeks)**	**4 predictors:** SFH3, diffSFH23, diffMAC23; MAC3*	**4 predictors:** SFH3, diffSFH23, diffMAC23; MAC3*	**11 predictors:** SFH3, MAC3*, WEIGHT3*, DBP3*, SBP3*, diffSFH23, diffMAC23, diffWEIGHT13, diffDBP23, diffWEIGHT23, diffSBP13

#Despite of the features selected by each rule of feature selection, five predictors of parity, maternal weight and maternal height at register, and maternal age, neonatal sex are additionally added into developing prediction models.

*These predictors additionally included into the final modelling are used to calculate the diffMAC23, diffSFH23, diffDBP23, diffDBP13, and diffSBP23.

Table S10. Validation performance of models by six machine learning algorithms in predicting preterm birth with the under-sampling method.

Models	Top 10% rule						Top 25% rule						Top 50% rule					
	5-CV	Testing set					5-CV	Testing set					5-CV	Testing set				
	Acc in training set	AUC (95% CI)	Acc	Sen	Spe	Optimal threshold	Acc in training set	AUC (95% CI)	Acc	Sen	Spe	Optimal threshold	Acc in training set	AUC (95% CI)	Acc	Sen	Spe	Optimal threshold
Early pregnancy models (< 18 gestational weeks)																		
CatBoost	0.547	0.588 (0.555, 0.621)	0.496	0.648	0.489	0.501	0.563	0.587 (0.553, 0.621)	0.539	0.614	0.536	0.504	0.564	0.575 (0.542, 0.607)	0.445	0.688	0.433	0.494
Random Forests	0.534	0.561 (0.529, 0.593)	0.540	0.571	0.539	0.514	0.524	0.576 (0.544, 0.607)	0.808	0.255	0.833	0.558	0.528	0.574 (0.542, 0.606)	0.663	0.440	0.673	0.520
Stacked model	0.541	0.561 (0.530, 0.594)	0.674	0.433	0.685	0.544	0.556	0.550 (0.518, 0.582)	0.791	0.255	0.816	0.617	0.526	0.524 (0.491, 0.557)	0.624	0.419	0.633	0.419
DNN	0.659	0.560 (0.526, 0.593)	0.366	0.752	0.349	0.444	0.804	0.515 (0.481, 0.549)	0.654	0.379	0.666	0.728	0.874	0.499 (0.466, 0.535)	0.545	0.483	0.548	0.619
SVM	0.720	0.575 (0.542, 0.608)	0.491	0.648	0.484	0.485	0.725	0.524 (0.492, 0.556)	0.340	0.745	0.322	0.498	0.720	0.515 (0.482, 0.548)	0.640	0.403	0.650	0.562
LR	0.549	0.579 (0.546, 0.612)	0.436	0.576	0.429	0.485	0.537	0.587 (0.553, 0.620)	0.493	0.517	0.492	0.498	0.542	0.572 (0.539, 0.606)	0.410	0.622	0.400	0.468
Middle pregnancy models (< 26 gestational weeks)																		
CatBoost	0.540	0.577 (0.543, 0.611)	0.474	0.664	0.465	0.499	0.546	0.577 (0.542, 0.611)	0.638	0.487	0.645	0.504	0.557	0.571 (0.537, 0.605)	0.490	0.634	0.483	0.499
Random Forests	0.526	0.566 (0.535, 0.598)	0.810	0.255	0.836	0.559	0.530	0.567 (0.536, 0.599)	0.857	0.188	0.888	0.570	0.542	0.575 (0.543, 0.606)	0.702	0.413	0.715	0.527
Stacked model	0.536	0.569 (0.537, 0.600)	0.778	0.285	0.801	0.559	0.530	0.561 (0.529, 0.593)	0.677	0.416	0.689	0.714	0.532	0.534 (0.484, 0.584)	0.278	0.844	0.254	0.322
DNN	0.667	0.536 (0.503, 0.569)	0.381	0.704	0.366	0.056	0.630	0.563 (0.530, 0.596)	0.541	0.574	0.540	0.515	0.598	0.497 (0.463, 0.531)	0.446	0.590	0.439	0.488
SVM	0.722	0.573 (0.539, 0.606)	0.497	0.641	0.491	0.490	0.727	0.580 (0.546, 0.613)	0.478	0.658	0.469	0.457	0.726	0.489 (0.457, 0.520)	0.251	0.819	0.225	0.500
LR	0.532	0.576 (0.543, 0.609)	0.460	0.547	0.456	0.490	0.559	0.582 (0.549, 0.615)	0.494	0.515	0.493	0.498	0.544	0.570 (0.537, 0.604)	0.417	0.612	0.408	0.468
Late pregnancy models (<37 gestational weeks)																		
CatBoost	0.592	0.679 (0.646, 0.712)	0.737	0.534	0.747	0.51	0.603	0.677 (0.644, 0.710)	0.675	0.604	0.679	0.512	0.602	0.681 (0.649, 0.714)	0.756	0.520	0.76	0.561

																7	
Random Forests	0.559	0.678 (0.651, 0.705)	0.904	0.238	0.935	0.643	0.584	0.677 (0.649, 0.704)	0.852	0.356	0.875	0.625	0.533	0.681 (0.654, 0.708)	0.882	0.302	0.909	0.646
Stacked model	1.000	0.641 (0.612, 0.670)	0.910	0.181	0.943	0.378	0.559	0.678 (0.650, 0.705)	0.860	0.352	0.883	0.630	0.552	0.672 (0.630, 0.714)	0.805	0.414	0.822	0.619
DNN	0.665	0.664 (0.631, 0.696)	0.700	0.537	0.707	0.567	0.665	0.656 (0.624, 0.689)	0.775	0.450	0.789	0.616	0.680	0.651 (0.619, 0.683)	0.655	0.584	0.658	0.536
SVM	0.740	0.673 (0.641, 0.706)	0.628	0.638	0.628	0.530	0.737	0.673 (0.640, 0.705)	0.690	0.570	0.696	0.586	0.735	0.668 (0.637, 0.700)	0.785	0.450	0.800	0.690
LR	0.607	0.675 (0.643, 0.707)	0.574	0.448	0.580	0.527	0.627	0.677 (0.645, 0.709)	0.635	0.386	0.646	0.555	0.589	0.662 (0.629, 0.394)	0.535	0.482	0.538	0.516

CV, cross validation; DNN, deep neural networks; SVM, support vector machine; LR, logistic regression. Acc, accuracy; Sen, sensitivity; Spec, specificity.

Table S11. The hyper-parameters of best-fitting model using CatBoost algorithm for predicting preterm birth.

Parameters	values
Depth	8
Learning rate	0.01
l2_leaf_reg	9
Iteration	500
rsm	0.95

rsm means the percentage of features to use at each split selection, when features are selected over again at random.

l2_leaf_reg means the coefficient at the L2 regularization term of the cost function.

Other parameters use their default values.

Table S12. SHAP values derived from the CatBoost-based late pregnancy model in the testing set.

Feature	mean	median	SD	Rank
DBP3	-0.034932494	-0.074576596	0.107985426	1
diffSFH23	-0.072897953	-0.06546453	0.190039564	2
diffMAC23	-0.070625275	-0.064611656	0.214086878	3
diffWEIGHT23	-0.034700647	-0.049884887	0.095853864	4
AST	-0.026924571	-0.032089489	0.035846682	5
SFH3	-0.100411969	-0.029988672	0.270254358	6
upper_mens	-0.010962718	-0.025858449	0.0383062	7
diffWEIGHT13	-0.015610314	-0.021538506	0.046140704	8
week24	-0.018628475	-0.02112218	0.043868411	9
diffSBP13	-0.014970605	-0.019250541	0.040926462	10
TBil	-0.014805118	-0.015520768	0.02582639	11
MAC2	-0.011509678	-0.015354579	0.029668554	12
hemoglobin	-0.019742815	-0.013809417	0.06070126	13
platelet	-0.012208525	0.012655438	0.086110333	14
regage	-0.025084266	-0.012203461	0.090316143	15
DBP2	-0.007395822	-0.011842774	0.025746032	16
WEIGHT3	-0.008323242	-0.010620323	0.017537794	17
m_height	-0.020444895	-0.009554976	0.093482658	18
diffWEIGHT12	-0.011916881	-0.009469007	0.045054463	19
MAC3	-0.008428814	-0.009093334	0.021193299	20
diffDBP23	-0.010083332	-0.007330512	0.02031118	21
neo_sex	-0.006721309	0.006839115	0.041970775	22
SBP3	-0.008602305	-0.006320813	0.045810748	23
beats	-0.009036399	-0.006217115	0.017627212	24
AIB	-0.006628016	-0.005037683	0.018422187	25
SBP2	-0.005646446	-0.004936837	0.02691548	26
BUN	-0.008706493	-0.004065651	0.038010245	27
para	-0.003289954	-0.003731523	0.011212382	28
m_weight	-0.003333658	-0.003682426	0.010494428	29
WEIGHT2	-0.003437302	-0.003681267	0.007775728	30
SBP1	-0.004236251	-0.002636382	0.016373547	31
SFH2	-0.005464382	-0.00250584	0.019859014	32
diffSBP23	-0.005069395	-0.002496868	0.021628497	33
WEIGHT1	0.000290714	-0.001727806	0.018978333	34
FBG	-0.004075235	0.001578027	0.025999528	35
menarche	-0.001405992	-0.001020149	0.009576497	36
leukocyte	-0.004745278	-0.000952793	0.026873371	37

SD, standard deviation.

Ranking is calculated by the median absolute Shapley values. The higher the median absolute Shapley values, the more importance the feature is associated with risk of preterm birth.

Table S13. Association of predictors in the best-fitting model with preterm birth in the cohort of 22,603 pregnancies.

Predictor	OR (95% CI)	P values
DBP3	1.04 (1.03,1.05)	<0.01
diffSFH23	0.84 (0.82,0.86)	<0.01
diffMAC23	0.92 (0.90,0.93)	<0.01
diffWEIGHT23	0.92 (0.90,0.94)	<0.01
AST	1.01 (1.00,1.02)	0.01
SFH3	0.81 (0.78,0.83)	<0.01
Length of a menstrual cycle, days	0.97 (0.95,1.00)	0.05
diffWEIGHT13	0.95 (0.93,0.97)	<0.01
Frequency of antenatal visits before 24 weeks		
< 2	1.00	
2 to 4	0.96 (0.70,1.30)	0.77
> 4	1.39 (1.03,1.89)	0.03
diffSBP13	1.01 (1.01,1.02)	<0.01
TBil	1.00 (0.98,1.02)	0.99
MAC2	1.02 (1.01,1.03)	<0.01
hemoglobin	1.01 (1.00,1.02)	<0.01
platelet	1.00 (1.00,1.00)	<0.01
regage	1.05 (1.04,1.07)	<0.01
DBP2	1.02 (1.01,1.03)	<0.01
WEIGHT3	1.00 (0.99,1.00)	0.31
m_height	0.97 (0.96,0.99)	<0.01
diffWEIGHT12	1.01 (0.98,1.03)	0.67
MAC3	0.99 (0.98,1)	0.02
diffDBP23	1.01 (1,1.02)	0.05
Neonatal sex		
Male	1.31 (1.15,1.49)	<0.01
Female	1.00	
SBP3	1.02 (1.01,1.03)	<0.01
beats	1.00 (0.99,1.01)	0.85
AIB	0.99 (0.97,1.01)	0.40
SBP2	1.01 (1.01,1.02)	<0.01
BUN	1.09 (1.01,1.19)	0.04
Parity		
0	1.00	
1	1.26 (1.11, 1.44)	<0.01
>1	1.77 (1.22, 2.58)	<0.01
Maternal weight	1.01 (1.00,1.02)	0.04
WEIGHT2	1.01 (1.00,1.02)	0.08
SBP1	1.01 (1.00,1.01)	0.05

SFH2	1.03 (1.00,1.05)	0.09
diffSBP23	1.00 (1.00,1.01)	0.43
WEIGHT1	1.01 (1.00,1.02)	0.11
FBG	1.10 (0.96,1.27)	0.18
Age at menarche	1.03 (0.98,1.09)	0.28
leukocyte	1.06 (1.02,1.09)	<0.01
